# Supplementary material for: Loss of NECTIN1 triggers melanoma dissemination upon local IGF1 depletion
Source: Nat Genet. 2022 Oct 13;54(12):1839–52. doi: 10.1038/s41588-022-01191-z (PMC9729115; doi:10.1038/s41588-022-01191-z)
Supplement: Supplementary file 1 — Supplementary Notes and Tables 1–4. [file 41588_2022_1191_MOESM1_ESM.pdf]

nature genetics

Article

https://doi.org/10.1038/s41588-022-01191-z

Loss of NECTIN1 triggers melanoma dissemination upon local IGF1 depletion

In the format provided by the authors and unedited

Supplementary Notes

Injection of a DNA vector targeting *nectin1a* and *nectin1b* into *Tg*(*mitf:BRAF*^{V600E}), *tp53*$^{-/-}$ zebrafish embryos allowed us to generate primary melanomas knockout for *nectin1*. Next-generation sequencing of *nectin1a* and *nectin1b* CRISPR loci in 65 individual primary zebrafish melanomas revealed that the average and median proportion of mutant alleles were 65.0 and 77.7 for *nectin1a*, and 75.9 and 85.2 for *nectin1b*, respectively (Fig. 2b). Given that not all cells are melanoma cells in a tumor, a proportion of mutant alleles greater than 75% likely corresponds to complete targeting, while a proportion comprised between 25 and 75% likely reflects partial gene inactivation, possibly mimicking heterozygous loss. We found 52% of tumors (34/65) with near-complete targeting (> 75% mutant alleles) in *nectin1a* versus 68% (44/65) in *nectin1b* (Fig. 2b). 40% of tumors (26/65) had near-complete targeting of both genes. 14% of tumors (9/65) had low or no targeting (< 25% mutant alleles) in *nectin1a* versus only 8% (5/65) in *nectin1b* (Fig. 2b). In 65 individual tumors, we detected a total of 124 and 70 different mutant alleles in *nectin1a* and *nectin1b*, respectively. The vast majority of CRISPR-induced insertions or deletions resulted in frameshifts. Furthermore, we observed that targeting *nectin1b* resulted in significantly lower allele diversity in each tumor than targeting *nectin1a*, with an average of 3.2 and 4.5 mutant alleles per tumor, respectively (median of 2 and 4 mutant alleles, respectively) (Fig. 2c). Examples of the nature and proportions of mutant alleles of *nectin1a* and *nectin1b* present in 10 representative individual primary zebrafish melanomas are shown in Figure 2d. Together, these data demonstrate a high gene targeting efficiency for our tissue-specific CRISPR system in primary zebrafish melanomas and further support a prominent role of *nectin1b* compared to *nectin1a* in the phenotypes observed *in vivo*.

The comparison of the mutational patterns of *nectin1a* and *nectin1b* between primary zebrafish melanomas, their corresponding allografts at the dorsal injection site, and disseminated patches, did not allow to assess the evolution of *nectin1b*-knockout clones upon transplantation or *in vivo* dissemination because of near-complete gene targeting in primary tumors (Extended data fig. 3b). Yet, it revealed some degree of clonal selection in disseminated patches originating from tumors with mosaic *nectin1a* targeting (Extended data fig. 3b), suggesting that our *in vivo* spreading assay offers favorable conditions for clone competition.

In primary zebrafish tumors targeting both *fak* and *nectin1*, next-generation sequencing of the *fak1a* and *fak1b* CRISPR loci demonstrated high gene targeting efficiency, with an average of 77% of *fak1a* and 63% of *fak1b* alleles being mutated, in par with the mutation rates detected for *nectin1* (Extended data fig. 8d). These results establish the ability of the CRISPR MiniCoopR vector system to generate compound knockout tumors in zebrafish.
