# Supplementary material for: Loss of NECTIN1 triggers melanoma dissemination upon local IGF1 depletion
Source: Nat Genet. 2022 Oct 13;54(12):1839–52. doi: 10.1038/s41588-022-01191-z (PMC9729115; doi:10.1038/s41588-022-01191-z)
Supplement: Supplementary file 2 — Reporting Summary [file 41588_2022_1191_MOESM2_ESM.pdf]

nature portfolio

Corresponding author(s): Julien Ablain

Last updated by author(s): Aug 21, 2022

Reporting Summary

Nature Portfolio wishes to improve the reproducibility of the work that we publish. This form provides structure for consistency and transparency in reporting. For further information on Nature Portfolio policies, see our Editorial Policies and the Editorial Policy Checklist.

Statistics

For all statistical analyses, confirm that the following items are present in the figure legend, table legend, main text, or Methods section.

n/a	Confirmed	
☐	☒	The exact sample size (*n*) for each experimental group/condition, given as a discrete number and unit of measurement
☐	☒	A statement on whether measurements were taken from distinct samples or whether the same sample was measured repeatedly
☐	☒	The statistical test(s) used AND whether they are one- or two-sided<br>*Only common tests should be described solely by name; describe more complex techniques in the Methods section.*
☒	☐	A description of all covariates tested
☐	☒	A description of any assumptions or corrections, such as tests of normality and adjustment for multiple comparisons
☐	☒	A full description of the statistical parameters including central tendency (e.g. means) or other basic estimates (e.g. regression coefficient) AND variation (e.g. standard deviation) or associated estimates of uncertainty (e.g. confidence intervals)
☐	☒	For null hypothesis testing, the test statistic (e.g. *F*, *t*, *r*) with confidence intervals, effect sizes, degrees of freedom and *P* value noted<br>*Give P values as exact values whenever suitable.*
☒	☐	For Bayesian analysis, information on the choice of priors and Markov chain Monte Carlo settings
☒	☐	For hierarchical and complex designs, identification of the appropriate level for tests and full reporting of outcomes
☐	☒	Estimates of effect sizes (e.g. Cohen's *d*, Pearson's *r*), indicating how they were calculated

Our web collection on statistics for biologists contains articles on many of the points above.

Software and code

Policy information about availability of computer code

Data collection	No software was used for data collection
Data analysis	FastQC version 0.11.9, Cutadapt version 2.0, Tophat version 2.0.11, Cufflinks version 2.2.1, GISTIC 2.0, Gene Ontology (GO Ontology database Released 2019-02-02) using PANTHER Overrepresentation Test (Released 20190308), Gene Set Enrichment Analysis GSEA 3.0 (released July 2017) using MSigDB version 6.2 (released July 2018), ImageJ version 1.51 (released 23 April 2018), and GraphPad Prism 9 softwares were used for data analysis. No custom code or software was use for any aspect of data processing or analysis.

For manuscripts utilizing custom algorithms or software that are central to the research but not yet described in published literature, software must be made available to editors and reviewers. We strongly encourage code deposition in a community repository (e.g. GitHub). See the Nature Portfolio guidelines for submitting code & software for further information.

Data

Policy information about availability of data

All manuscripts must include a data availability statement. This statement should provide the following information, where applicable:

- Accession codes, unique identifiers, or web links for publicly available datasets
- A description of any restrictions on data availability
- For clinical datasets or third party data, please ensure that the statement adheres to our policy

The Cancer Genome Atlas (TCGA) dataset for cutaneous melanoma used in this study is available at https://gdc.cancer.gov/about-data/publications/pancanatlas. Copy-number analyses were performed with GISTIC 2.0 through the interface of the Broad Institute (https://portals.broadinstitute.org/tcga/home), using 2014-04-28 stddata release.
The RNA sequencing data of the consequences of NECTIN1 loss and the effect of serum depletion on A375 human melanoma cells generated during this study have

been deposited in GEO under the accession code GSE174150 and are publicly available.
The tandem mass spectrometry data of the cell-surface proteins of NECTIN1-deficient and NECTIN1-proficient A375 human melanoma cells upon serum depletion generated during this study have been deposited in MassIVE (Center for Computational Mass Spectrometry, UCSD) under the accession code MSV000090170 and are publicly available.

Field-specific reporting

Please select the one below that is the best fit for your research. If you are not sure, read the appropriate sections before making your selection.

☒ Life sciences ☐ Behavioural & social sciences ☐ Ecological, evolutionary & environmental sciences

For a reference copy of the document with all sections, see nature.com/documents/nr-reporting-summary-flat.pdf

Life sciences study design

All studies must disclose on these points even when the disclosure is negative.

Sample size
For cell line work, no calculation was performed to predetermine sample size; experiments were repeated four times to ensure robustness, unless statistical significance could be reached with 3 replicates only. For zebrafish transplantation experiments, a target sample size of 16 primary tumors per arm was calculated such that 2 fold differences in tumor spreading could be observed with a power of 80% and with statistical significance (5% type I error) using two-sided student's t-test. This sample size was never attained because statistical significance was reached with smaller samples due to the larger size of the observed effects. For human melanoma sections, sample size was limited by availability.

Data exclusions
No data were excluded from the analyses.

Replication
All experiments were independently repeated at least times and the aggregated data reported in the manuscript include all replicates. All attempts at replication were successful for experiments presented in the manuscript and that showed statistically significant differences.

Randomization
For injection and transplantation experiments in zebrafish, embryos and secondary recipients were randomly allocated to each genetic condition. For cell culture experiments implicating genetic or pharmacological modifications, cells were randomly distributed between the different experimental conditions. Randomization was irrelevant to all other experiments, which did not involve allocation of samples or organisms to different experimental groups.

Blinding
Data collection was performed blinded in the case of tumor-initiation and transplantation experiments in zebrafish, as well as for immunofluorescence, immunohistochemistry and H&E staining of human or zebrafish tissue-sections. In the case of cell culture experiments, blinding was performed whenever possible, depending on research personnel availability. Blinding was only lifted at the time of data analysis.

Reporting for specific materials, systems and methods

We require information from authors about some types of materials, experimental systems and methods used in many studies. Here, indicate whether each material, system or method listed is relevant to your study. If you are not sure if a list item applies to your research, read the appropriate section before selecting a response.

Materials & experimental systems

n/a	Involved in the study	
☐	☒	Antibodies
☐	☒	Eukaryotic cell lines
☒	☐	Palaeontology and archaeology
☐	☒	Animals and other organisms
☐	☒	Human research participants
☒	☐	Clinical data
☒	☐	Dual use research of concern

Methods

n/a	Involved in the study	
☒	☐	ChIP-seq
☒	☐	Flow cytometry
☒	☐	MRI-based neuroimaging

Antibodies

Antibodies used
All antibodies used in this study are reported in the Methods section.
NECTIN1 primary antibody (HPA026846, Sigma-Aldrich) , dilution 1:50 for IHC, 1:200 for IF
goat-anti-rabbit HRP secondary antibody (PI-1000, Vector Laboratories) , dilution 1:200 for IHC
Phospho-Histone3 (Ser10) primary antibody (#9701, Cell Signaling Technology), dilution 1:200 for IHC
Phospho-FAK (Tyr397) primary antibody (44-624G, Invitrogen), dilution 1:200 for IHC
α-E-Catenin primary antibody (#3236, Cell Signaling Technology), dilution 1:200 for IF
N-Cadherin primary antibody (#13116, Cell Signaling Technology), dilution 1:200 for IF
α-Actinin primary antibody (#69758, Cell Signaling Technology), dilution 1:100 for IF
α-Catenin primary antibody (13-9700, Invitrogen), dilution 1:200 for IF
Phospho-IGF1R beta (Tyr1161) primary antibody (PA5-37601, Invitrogen), dilution 1:200 for IF

E-Cadherin (4A2) primary antibody (#14472, Cell Signaling Technology), dilution 1:200 for IF
Alexa Fluor 488 goat anti-rabbit secondary antibody (A11008, Life Technologies), dilution 1:400 for IF
Alexa Fluor 568 goat anti-mouse secondary antibody (A11004, Life Technologies), dilution 1:400 for IF
Alexa Fluor Plus 488 Goat anti-Mouse secondary antibody (A32723, Life Technologies), dilution 1:400 for IF
Alexa Fluor 568 Goat anti-Rabbit secondary antibody (A11036, Life Technologies), dilution 1:400 for IF
NECTIN1 primary antibody (ab66985, Abcam), dilution 1:1000 for WB
phospho-FAK primary antibody (#3283, Cell Signaling Technology), dilution 1:1000 for WB
FAK primary antibody (#71433, Cell Signaling Technology), dilution 1:1000 for WB
phospho-SRC primary antibody (#2101, Cell Signaling Technology), dilution 1:1000 for WB
SRC primary antibody (#2109, Cell Signaling Technology), dilution 1:1000 for WB
ACTIN primary antibody (A2228, Sigma-Aldrich), dilution 1:5000 for WB
anti-mouse HRP (#7076, Cell Signaling Technology), dilution 1:2000 for WB
anti-rabbit HRP (#7074, Cell Signaling Technology), dilution 1:2000 for WB
Fibronectin (#26836, Cell Signaling Technology), dilution 1:400 for IF
Laminin (L9393, Sigma-Aldrich), dilution 1:100 for IF
FNDC3A (PA5-109309, Invitrogen) , dilution 1:200 for IF
Integrin β1 (#9699, Cell Signaling Technology), dilution 1:1000 for WB
Integrin β2 (#73663, Cell Signaling Technology), dilution 1:1000 for WB
Integrin β3 (#13166, Cell Signaling Technology), dilution 1:1000 for WB
Integrin β4 (#14803, Cell Signaling Technology), dilution 1:1000 for WB
Integrin β5 (#3629, Cell Signaling Technology), dilution 1:1000 for WB
GAPDH (ab8245, Abcam), dilution 1:1000 for WB
CD49f (Integrin alpha 6) (14-0495-85, eBioscience), 40ug/mL for inhibition

Validation

All Cell Signaling Technology and Invitrogen antibodies were validated for use in human samples by the companies, as described on the suppliers' websites. NECTIN1 primary antibody is a Prestige Antibody developed and validated by the Human Protein Atlas (HPA) project and characterization data is accessible via the Human Protein Atlas portal. We validated NECTIN1 primary antibody (ab66985, Abcam) by knockdown and overexpression experiments as shown in Extended Data Fig. 3g. We validated Phospho-FAK (Tyr397) primary antibody (44-624G, Invitrogen) for use in zebrafish by comparing IHC staining in fak wild-type and knockout zebrafish tumors. Laminin (L9393, Sigma-Aldrich) and FNDC3A (PA5-109309, Invitrogen) primary antibodies were validated by other groups for use in zebrafish as documented on the ZFIN website (https://zfin.org/ZDB-ATB-090304-3#summary and https://zfin.org/ZDB-ATB-200313-1#summary).

Eukaryotic cell lines

Policy information about cell lines

Cell line source(s)

293T, RPMI-7951, CJM, A375, SKMEL2, COLO679, and G-361 cell lines were purchased from commercial sources (ATCC CRL-3216, ATCC HTB-66, Creative Bioarray CSC-C6421J, ATCC CRL-1619, ATCC HTB-68, Sigma 87061210, and ATCC CRL-1424, respectively)

Authentication

The cell lines used in this study were purchased directly from vendors that certify their authenticity but we did not authenticate them ourselves.

Mycoplasma contamination

All cell lines were tested negative for mycoplasma contamination throughout the study.

Commonly misidentified lines
(See ICLAC register)

No commonly misidentified cell lines were used in the study.

Animals and other organisms

Policy information about studies involving animals; ARRIVE guidelines recommended for reporting animal research

Laboratory animals

Zebrafish (Danio rerio) of the casper (mitfa-/-, roy-/-) and Tg(mitfa:BRAFV600E), tp53-/-, mitfa-/- strains were used in this study. Both males and females were used between 3 and 6 months of age.

Wild animals

The study did not involve wild animals.

Field-collected samples

The study did not involve samples collected from the field.

Ethics oversight

Zebrafish were handled according to our vertebrate animal protocol that has been approved by Boston Children's Hospital Animal Care Committee.

Note that full information on the approval of the study protocol must also be provided in the manuscript.

Human research participants

Policy information about studies involving human research participants

Population characteristics

Since all tissue samples used in this study were deidentified archived samples, no information about subjects was available.

Recruitment

Human melanoma tissue-sections were obtained from archived cases of the Brigham and Women's Hospital and patients were not recruited specifically for this study.

Ethics oversight	The human melanoma tissue-sections were used in this study with approval of the Institutional Review Board of Brigham and Women's Partners Human Research Committee, Harvard Medical School.

Note that full information on the approval of the study protocol must also be provided in the manuscript.
