# Supplementary figures and images for: Loss of NECTIN1 triggers melanoma dissemination upon local IGF1 depletion
Source: Nat Genet. 2022 Oct 13;54(12):1839–52. doi: 10.1038/s41588-022-01191-z (PMC9729115; doi:10.1038/s41588-022-01191-z)

# ITGB1

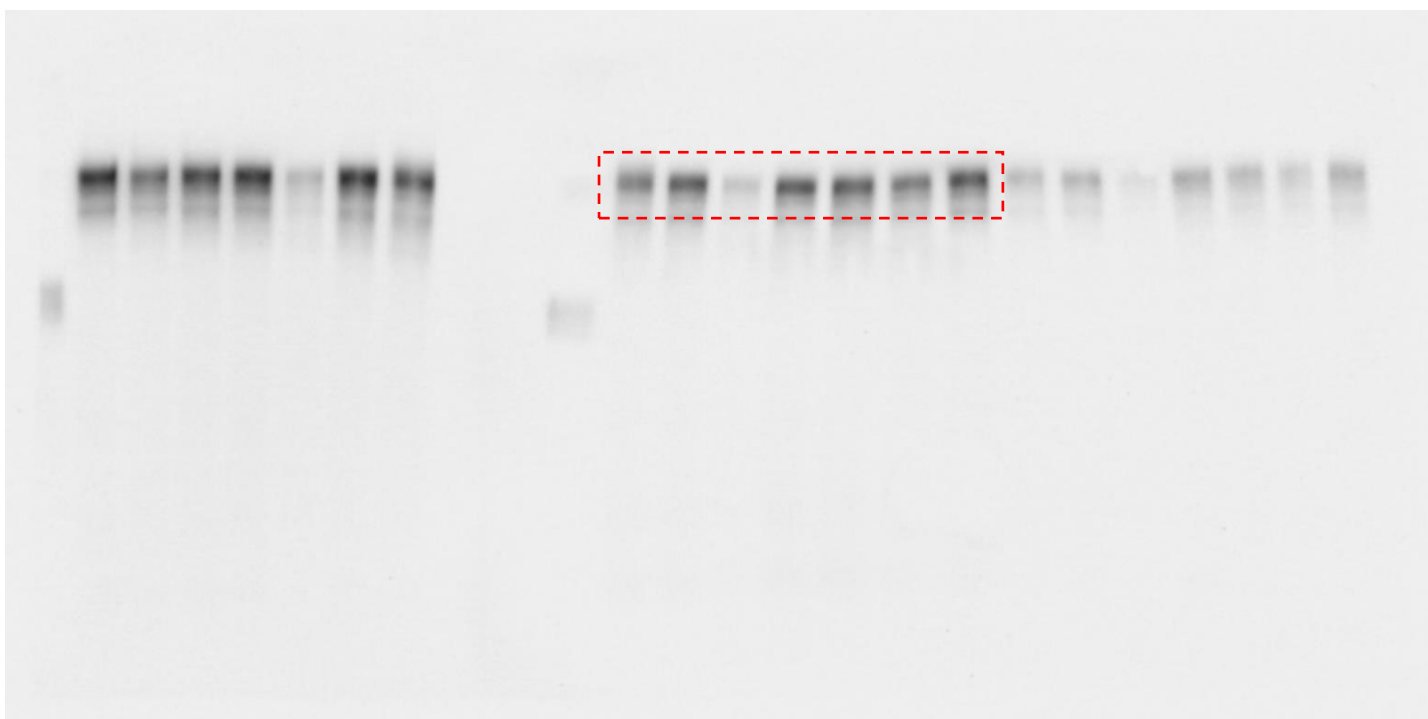

## Colorimetric

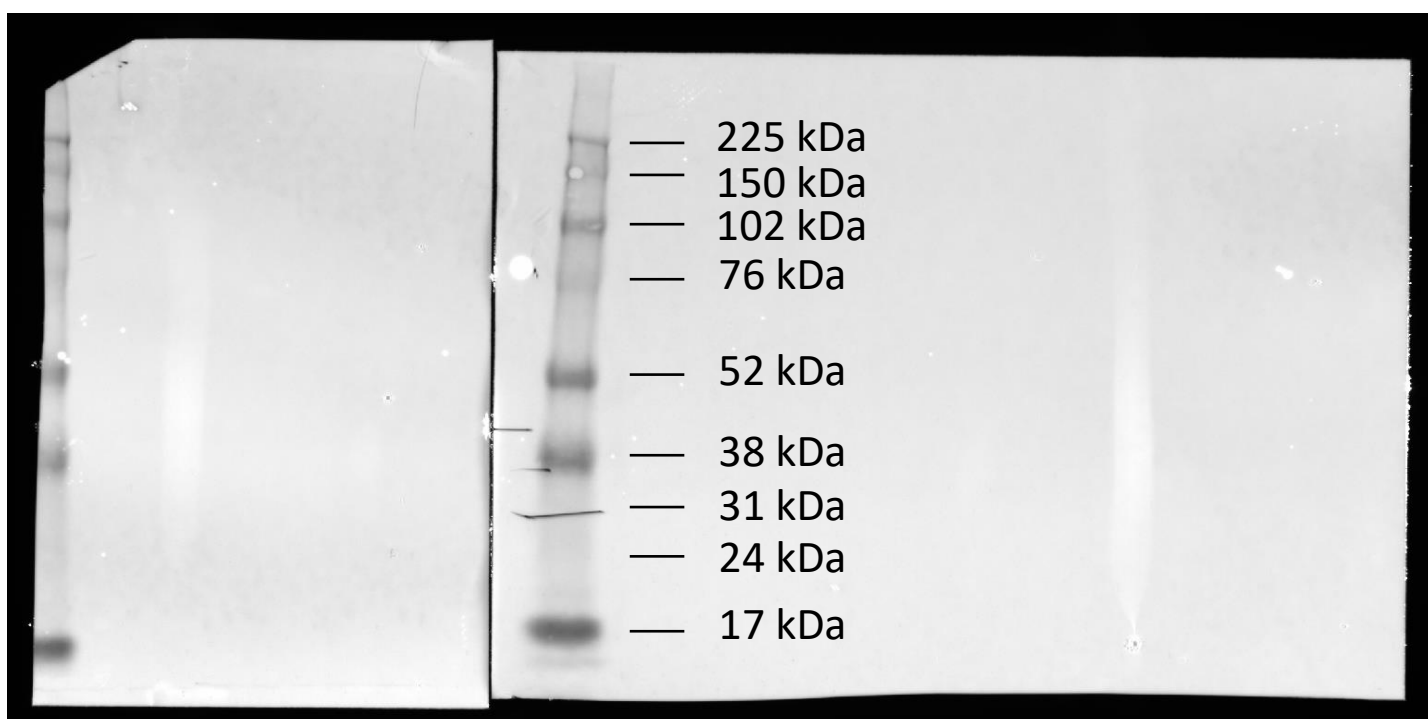

ITGB5

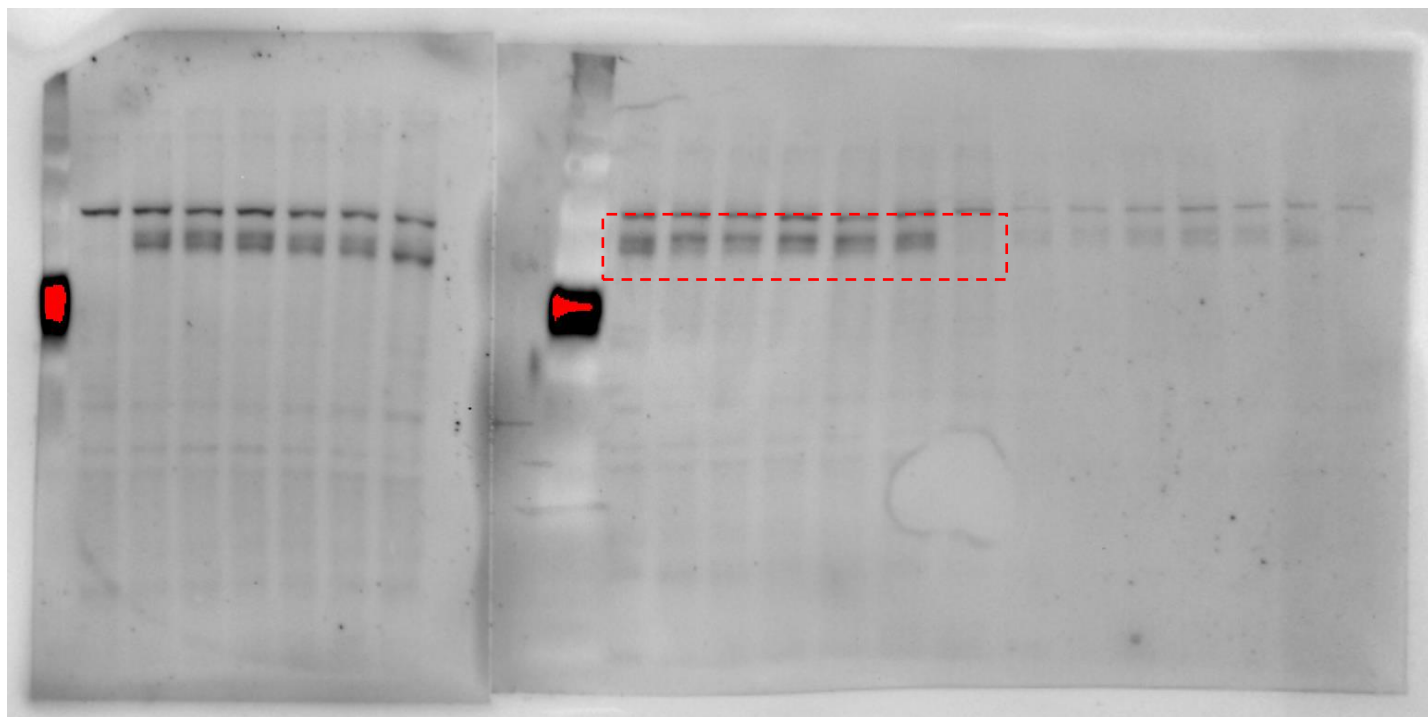

GAPDH

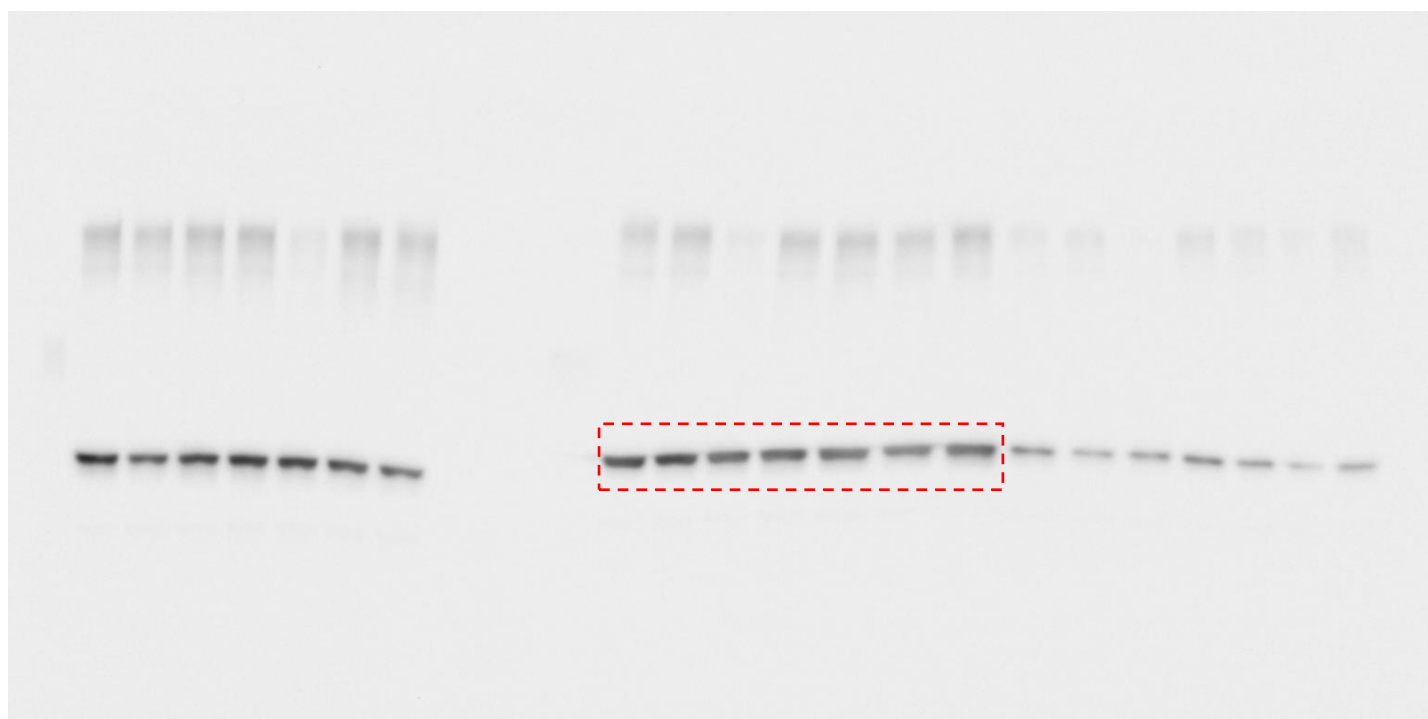

ITGB2

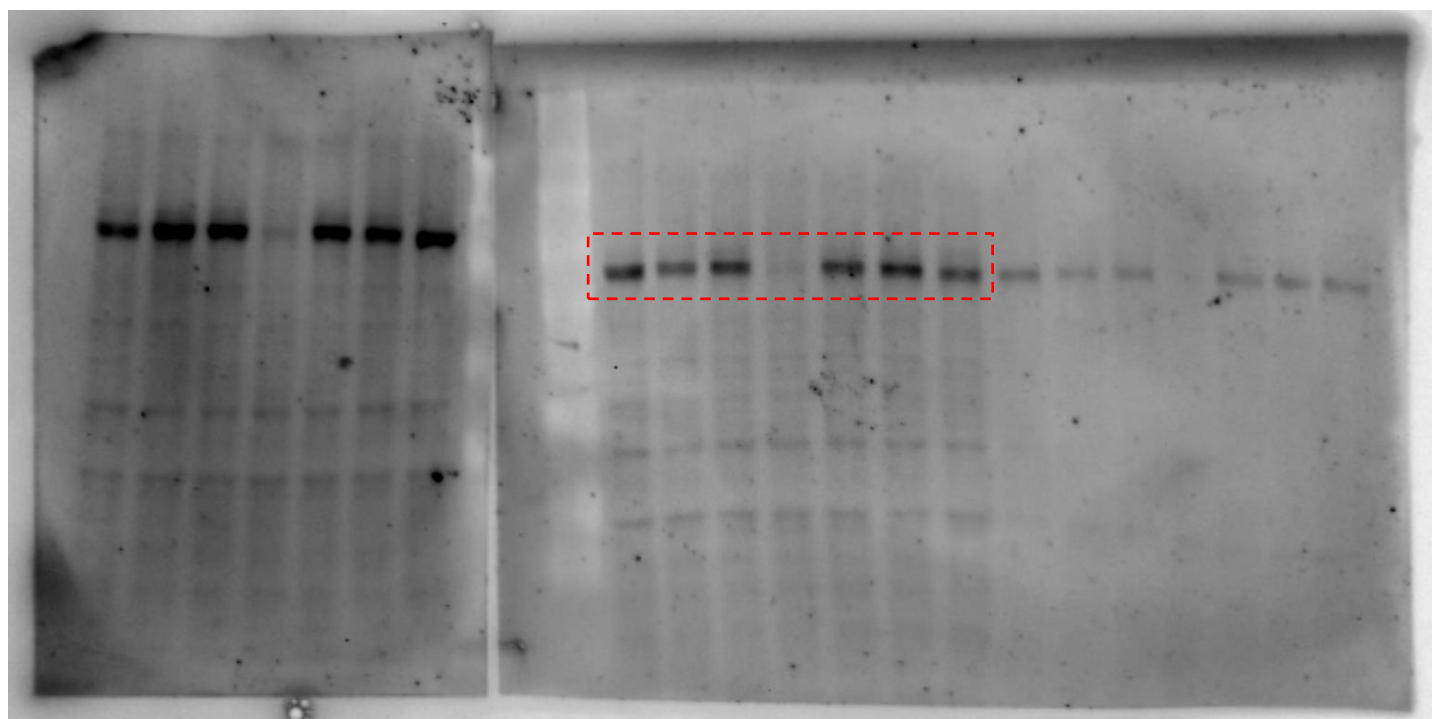

ITGB3

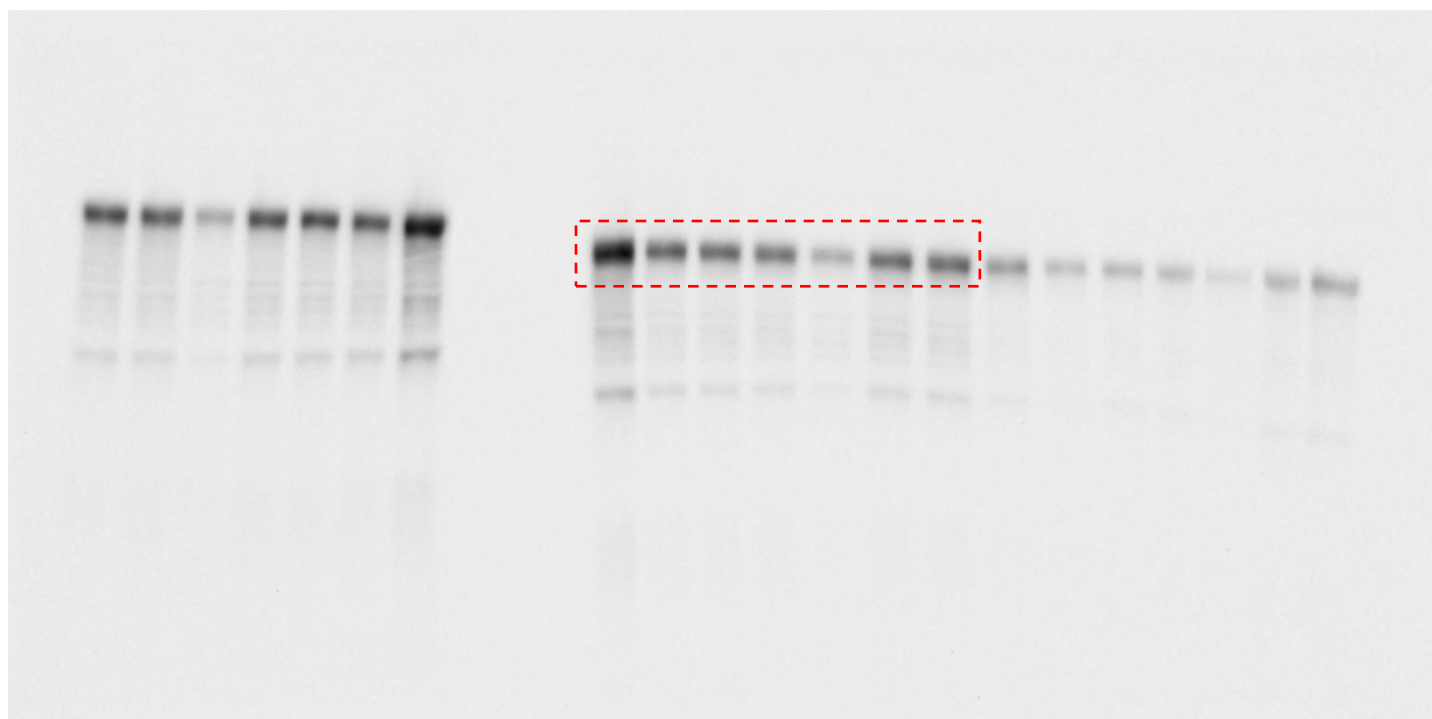

# ITGB4

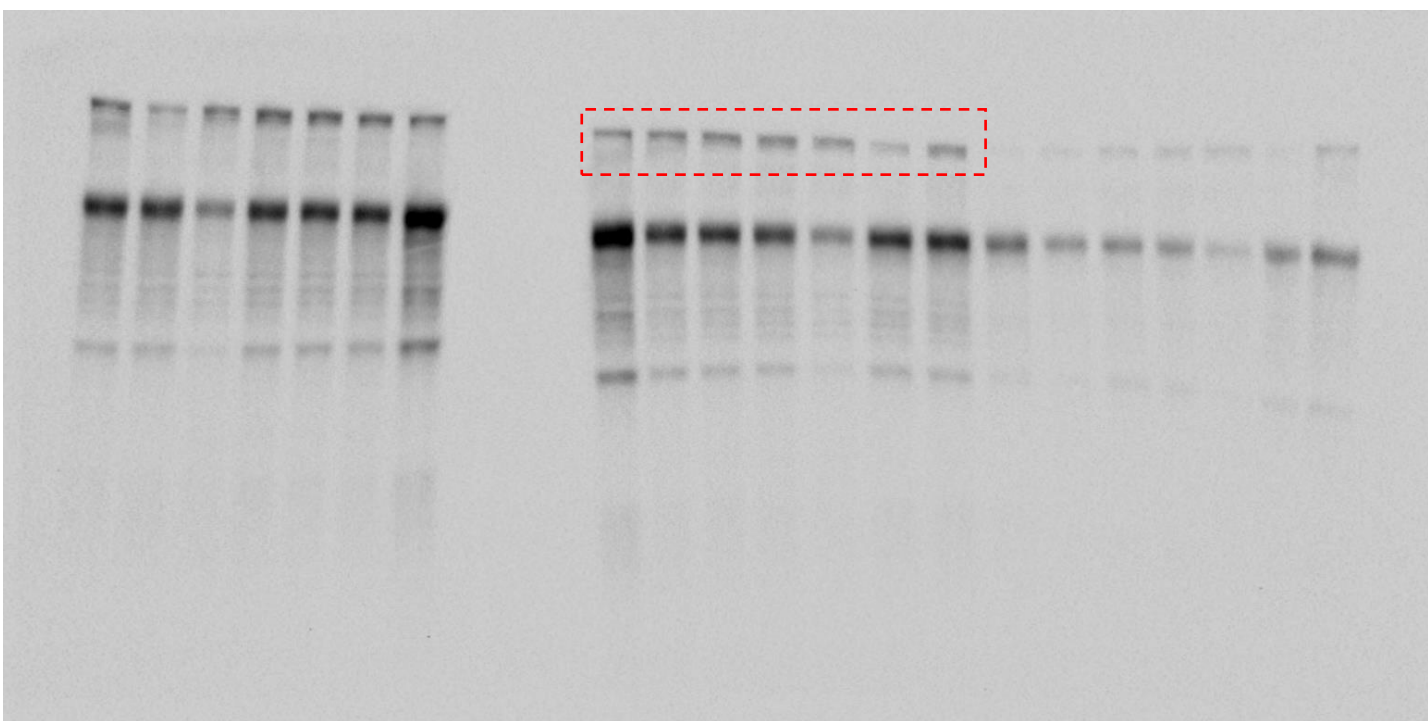

## Colorimetric

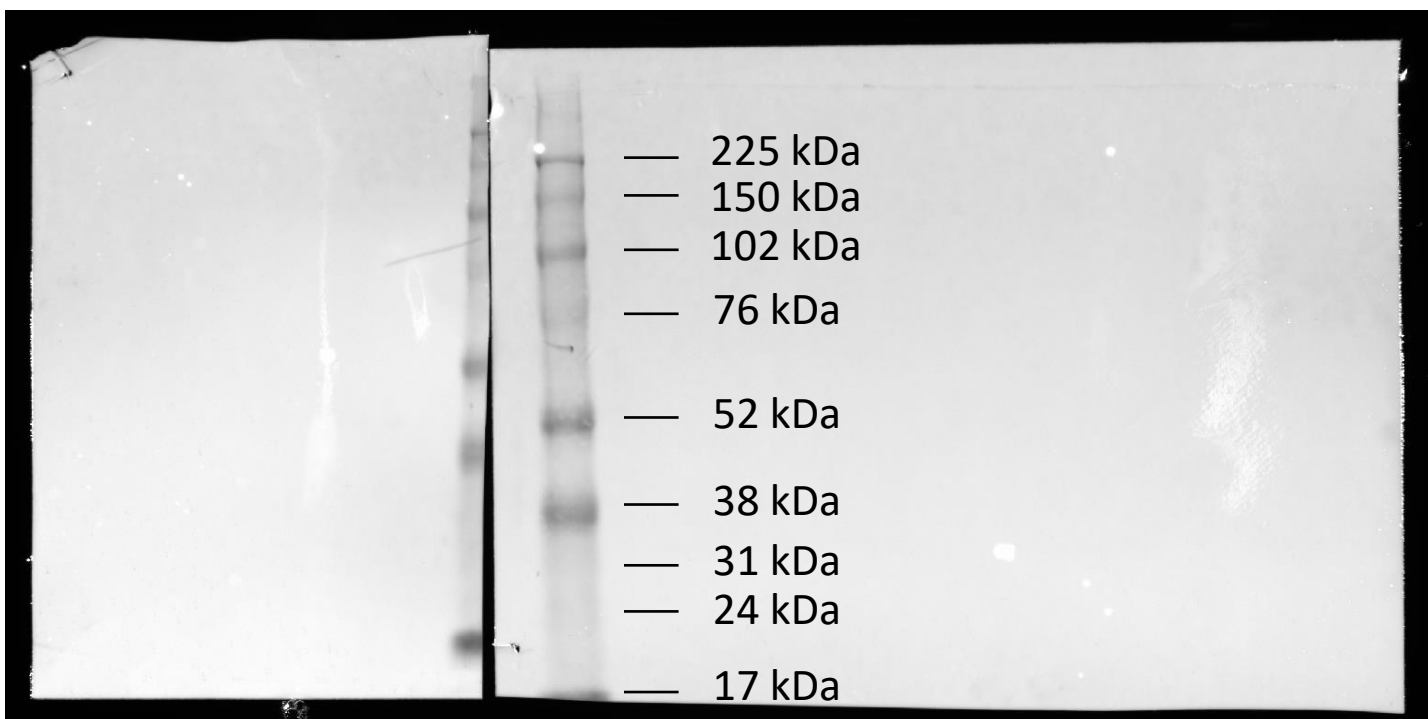

Supplement: Source Data Fig. 5 — Unprocessed western blots. [file 41588_2022_1191_MOESM10_ESM.pdf]

p-FAK

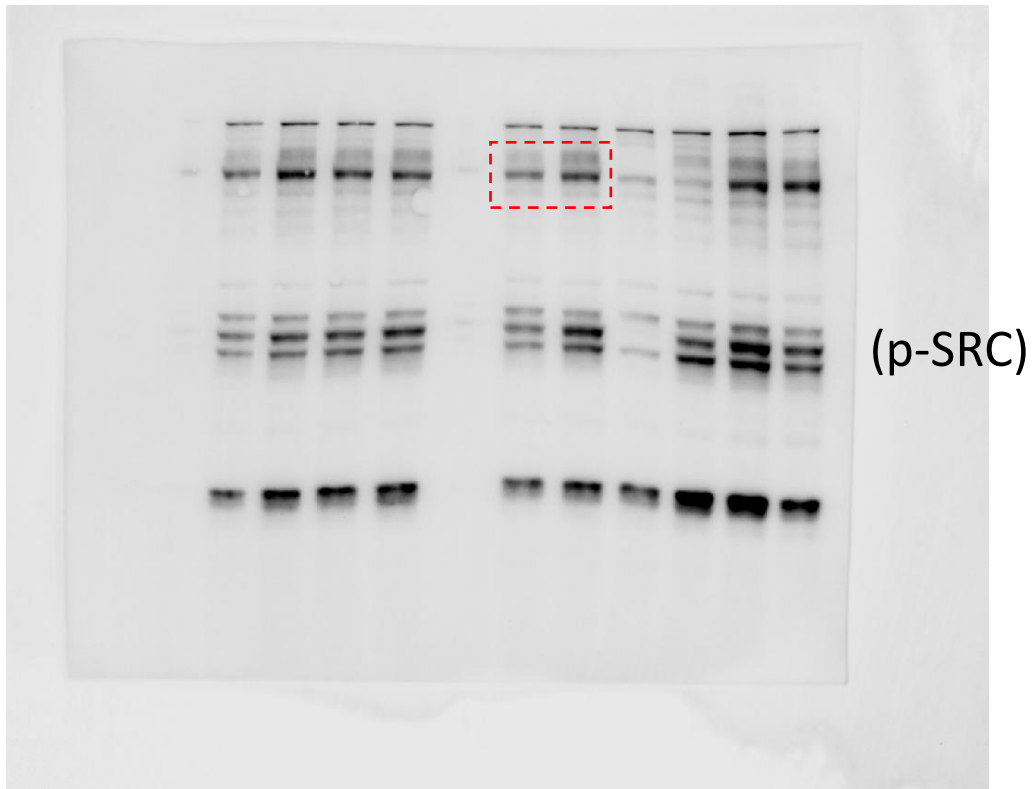

FAK

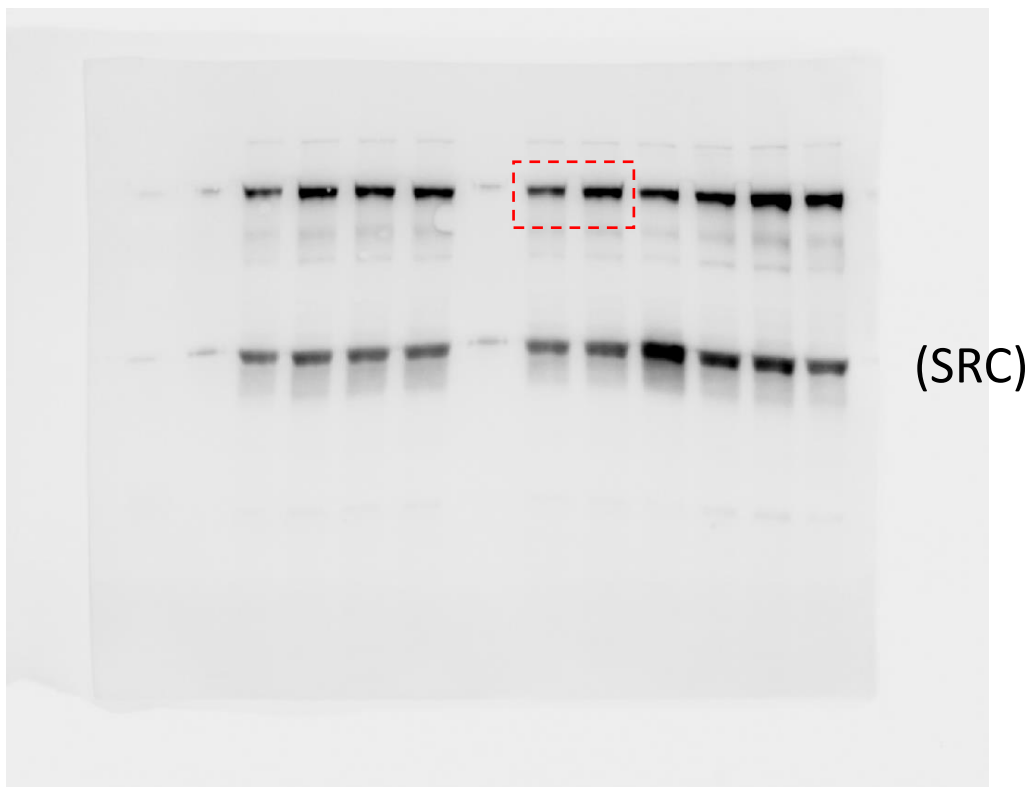

p-SRC

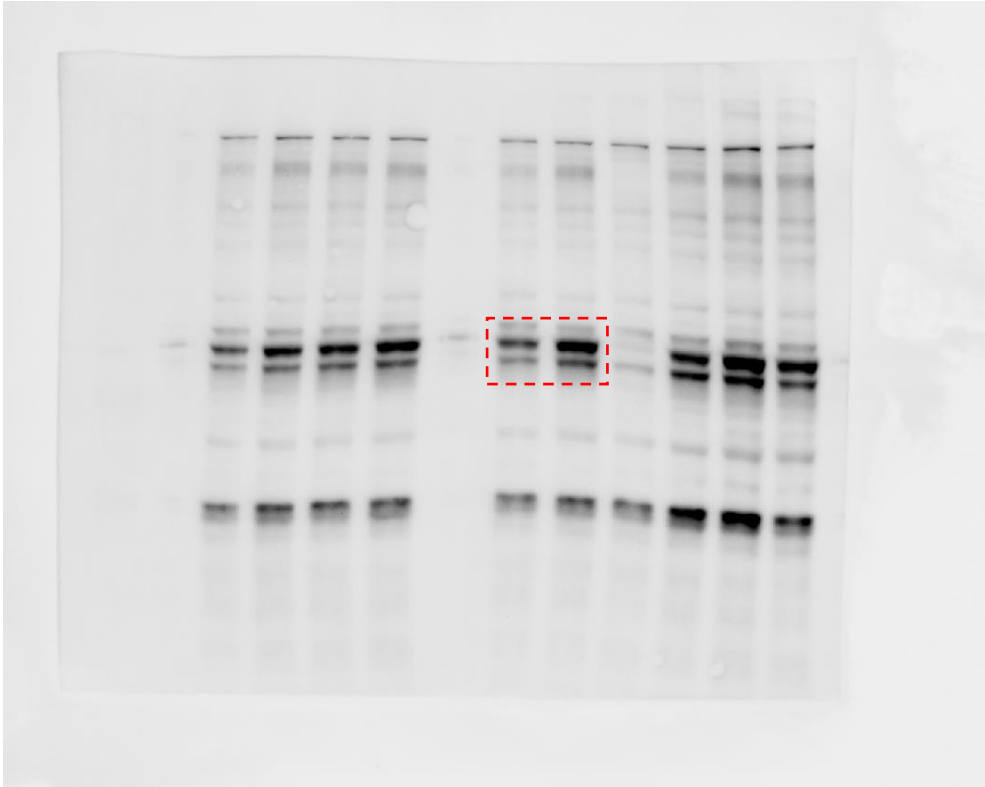

SRC

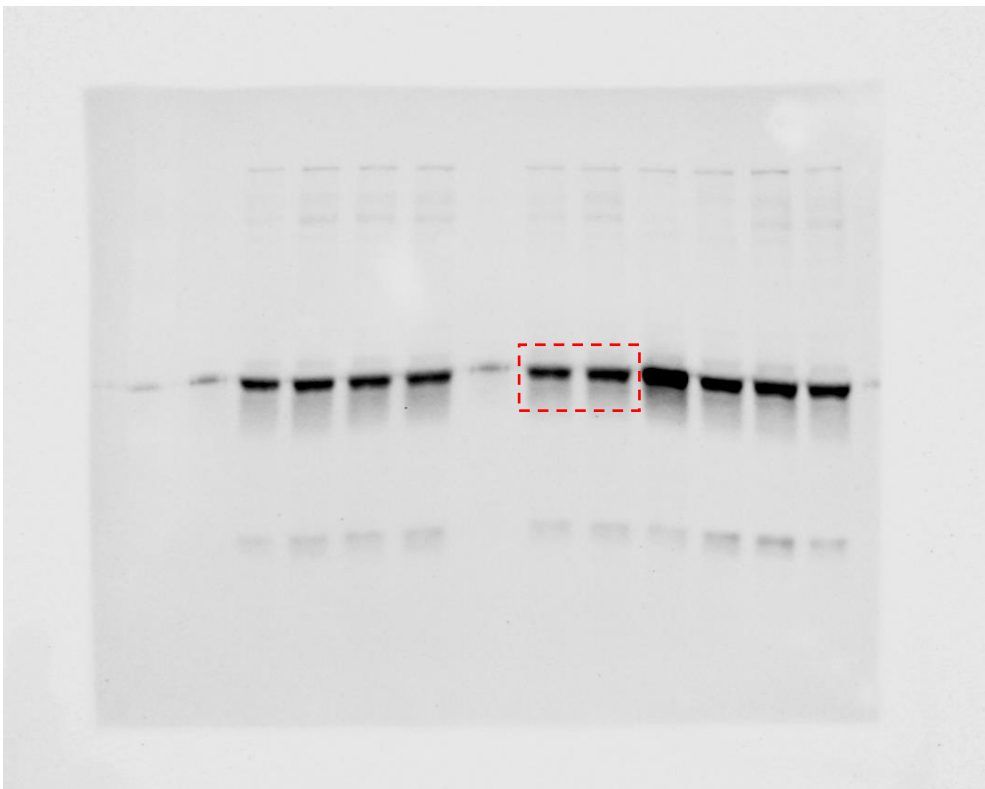

# Colorimetric

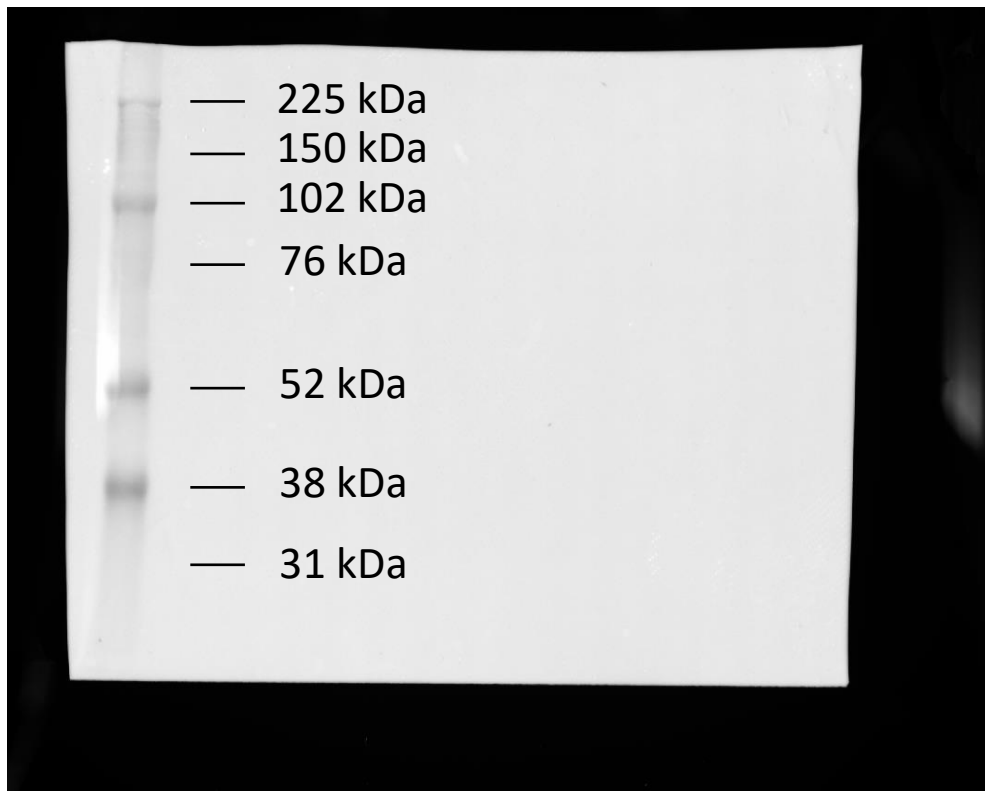

## GAPDH

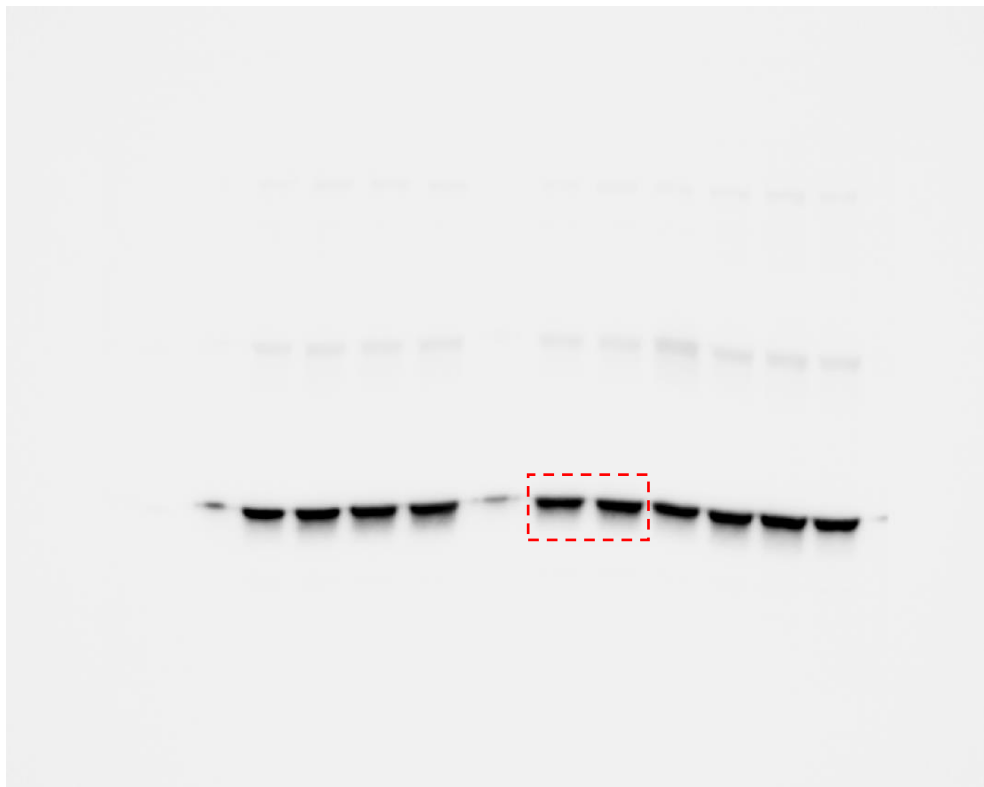

Supplement: Source Data Fig. 6 — Unprocessed western blots. [file 41588_2022_1191_MOESM12_ESM.pdf]

Extended Data Fig 3g

ACTIN

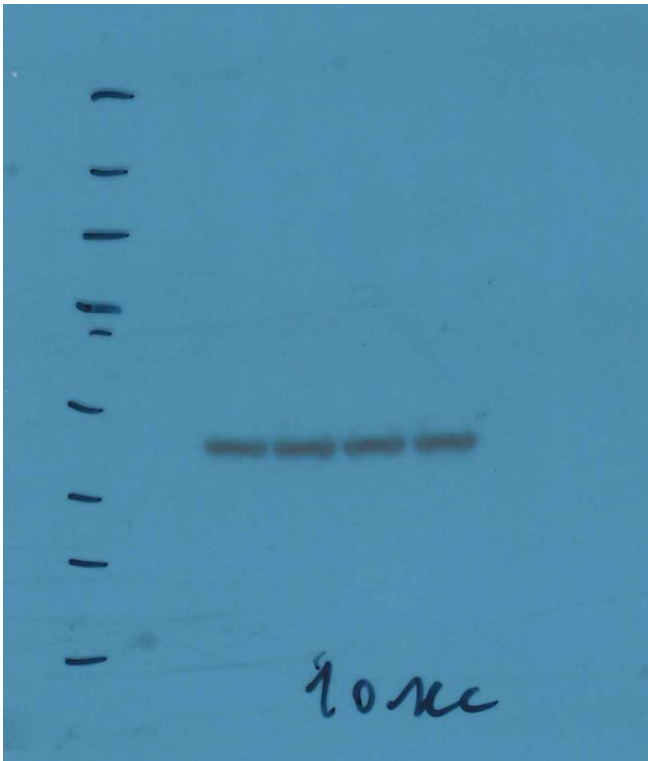

NECTIN1

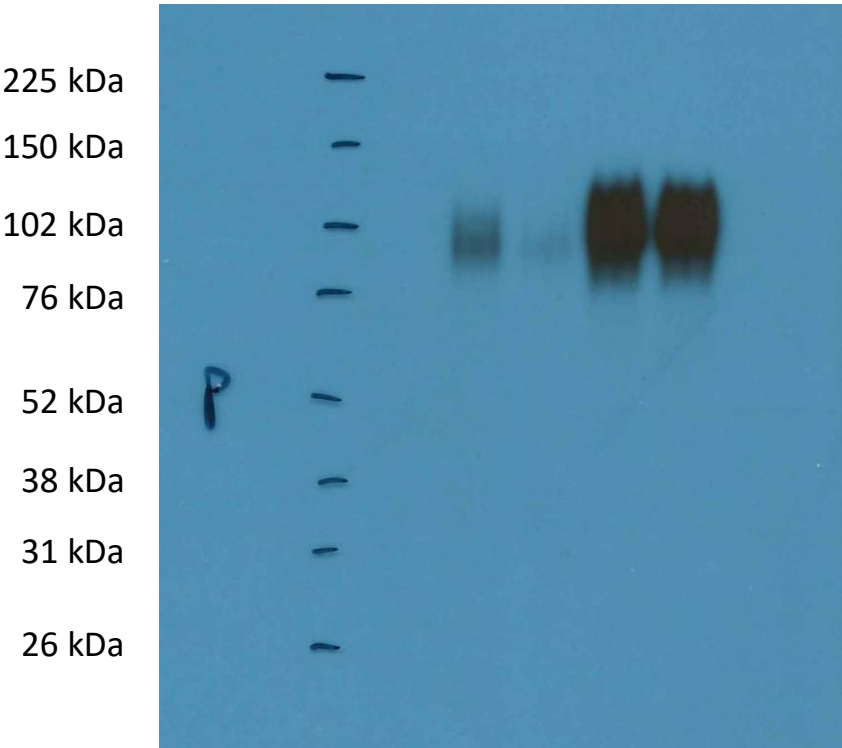

Extended Data Fig 3i

NECTIN1

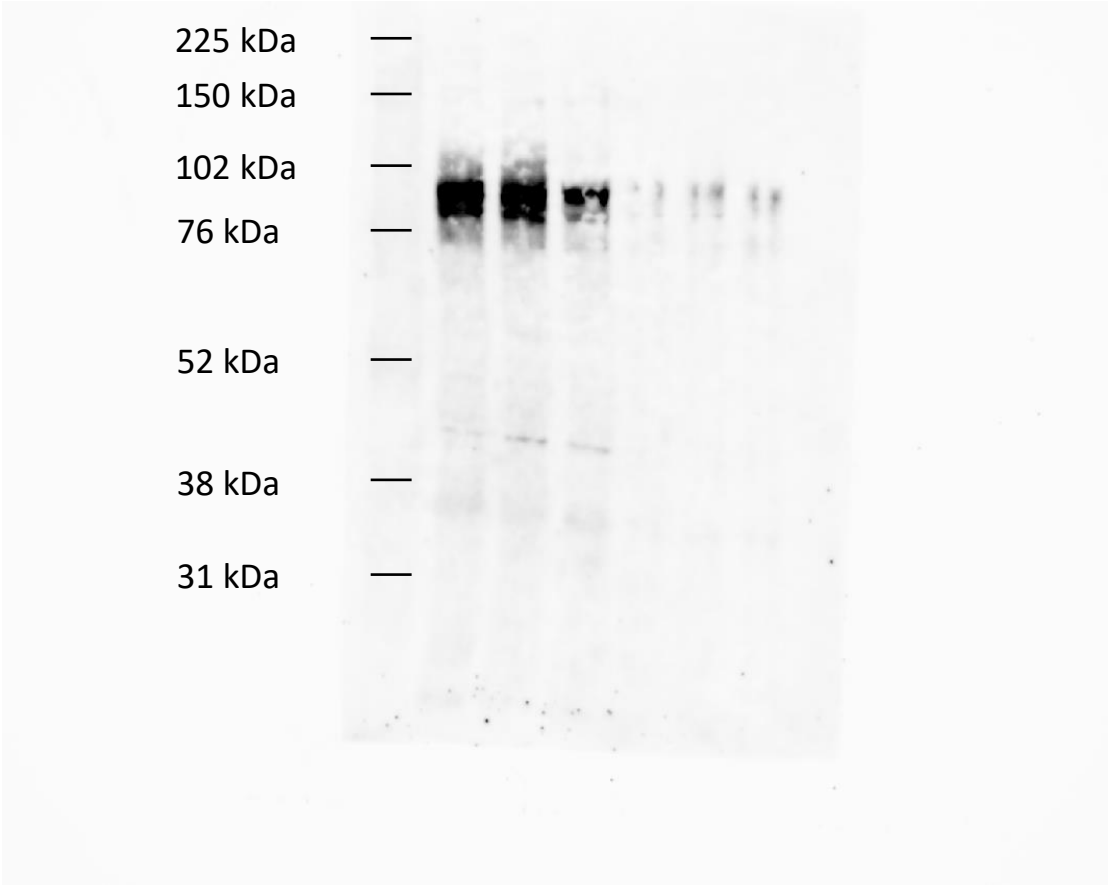

ACTIN

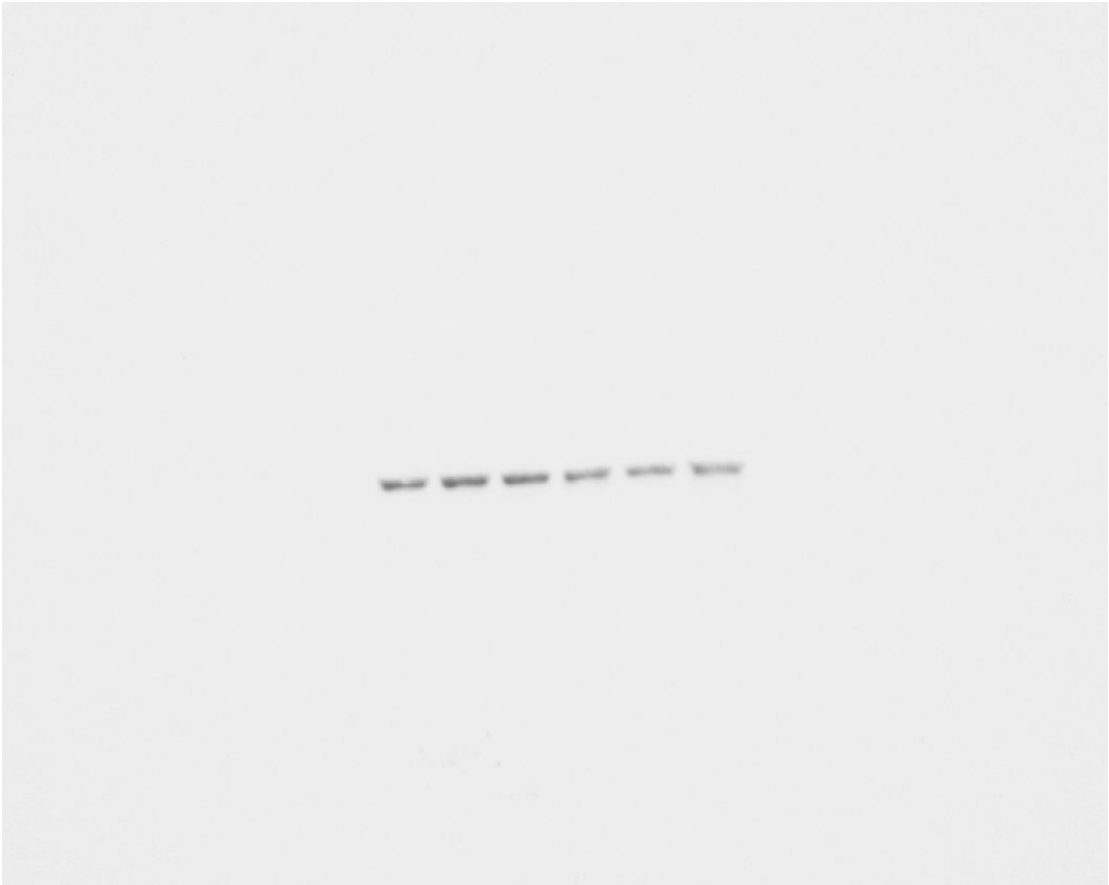

Supplement: Source Data Extended Data Fig. 3 — Unprocessed western blots. [file 41588_2022_1191_MOESM18_ESM.pdf]

# NECTIN1

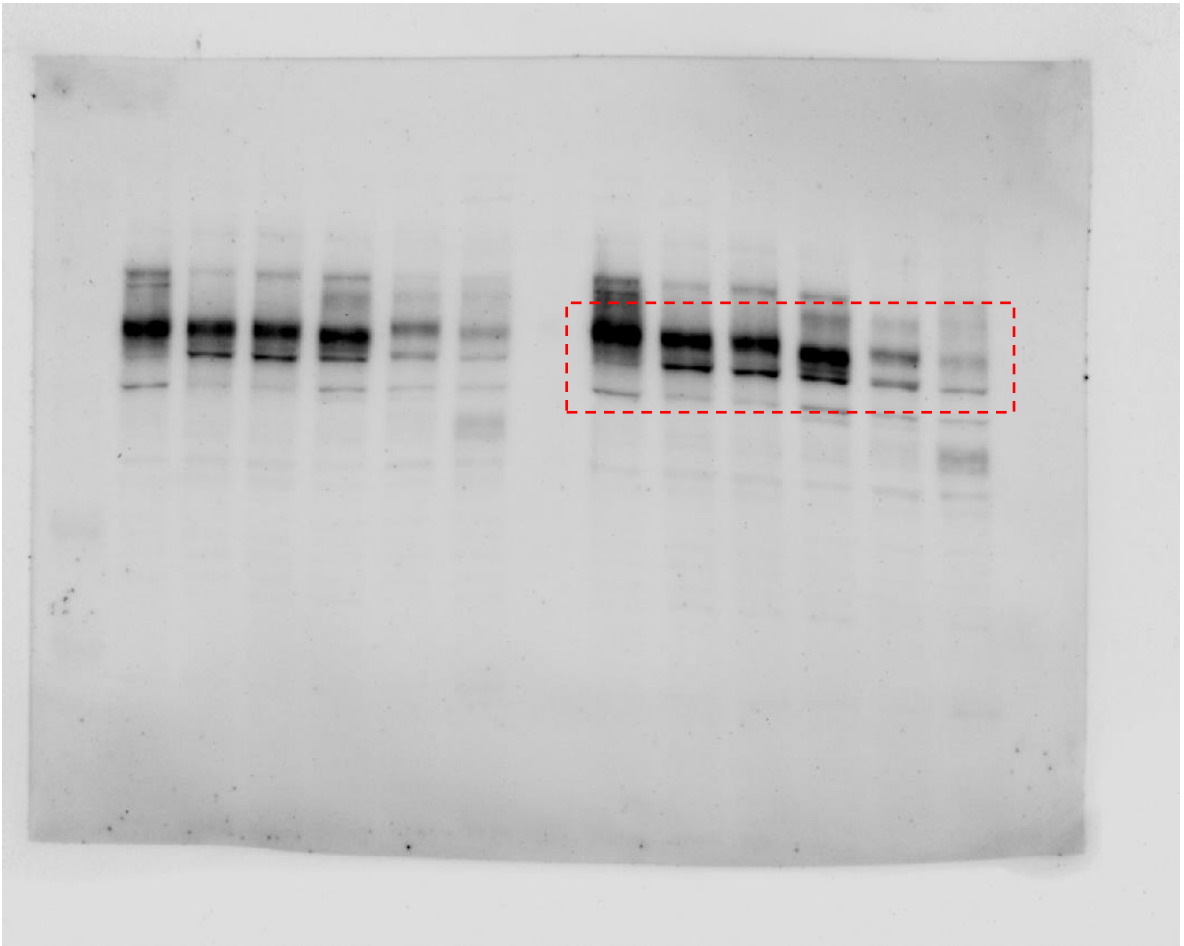

## Colorimetric

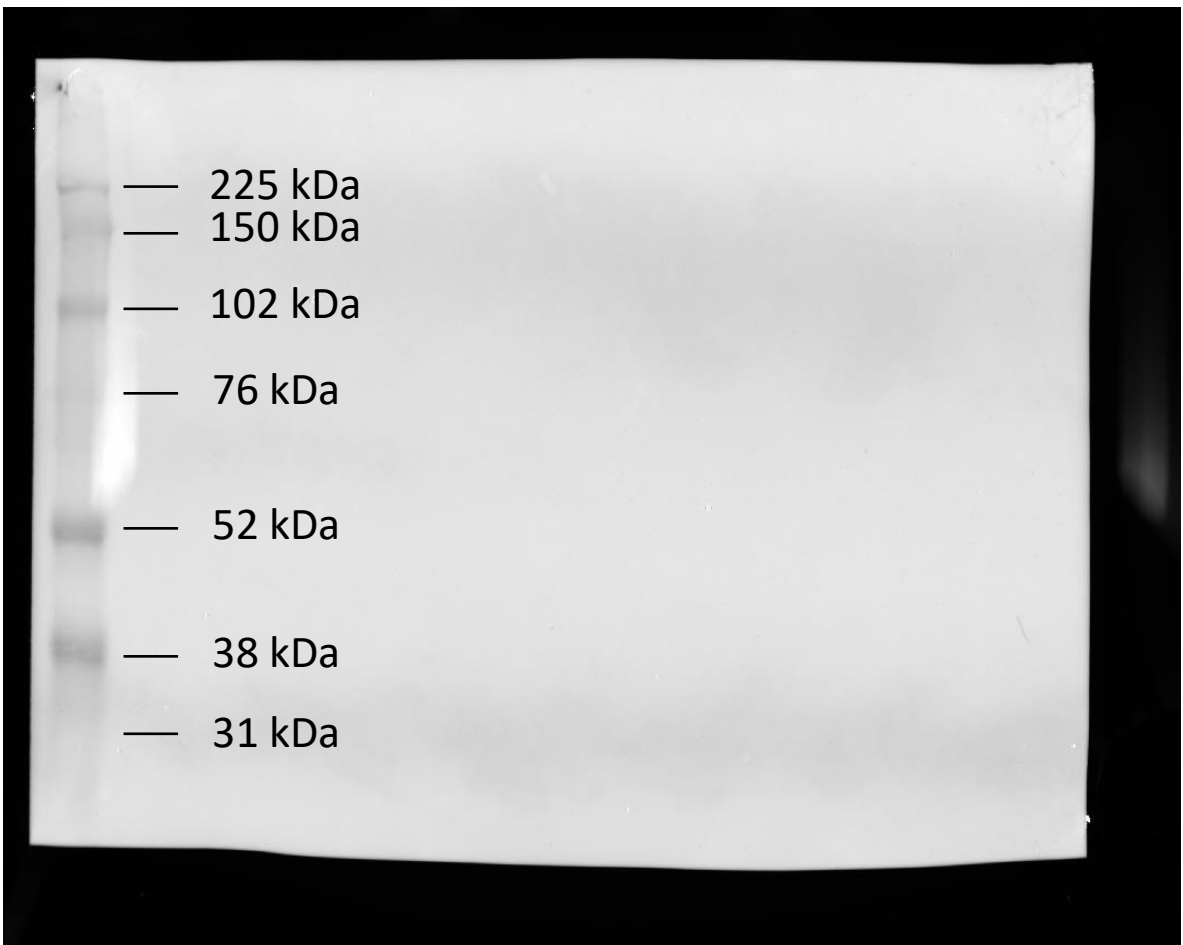

GAPDH

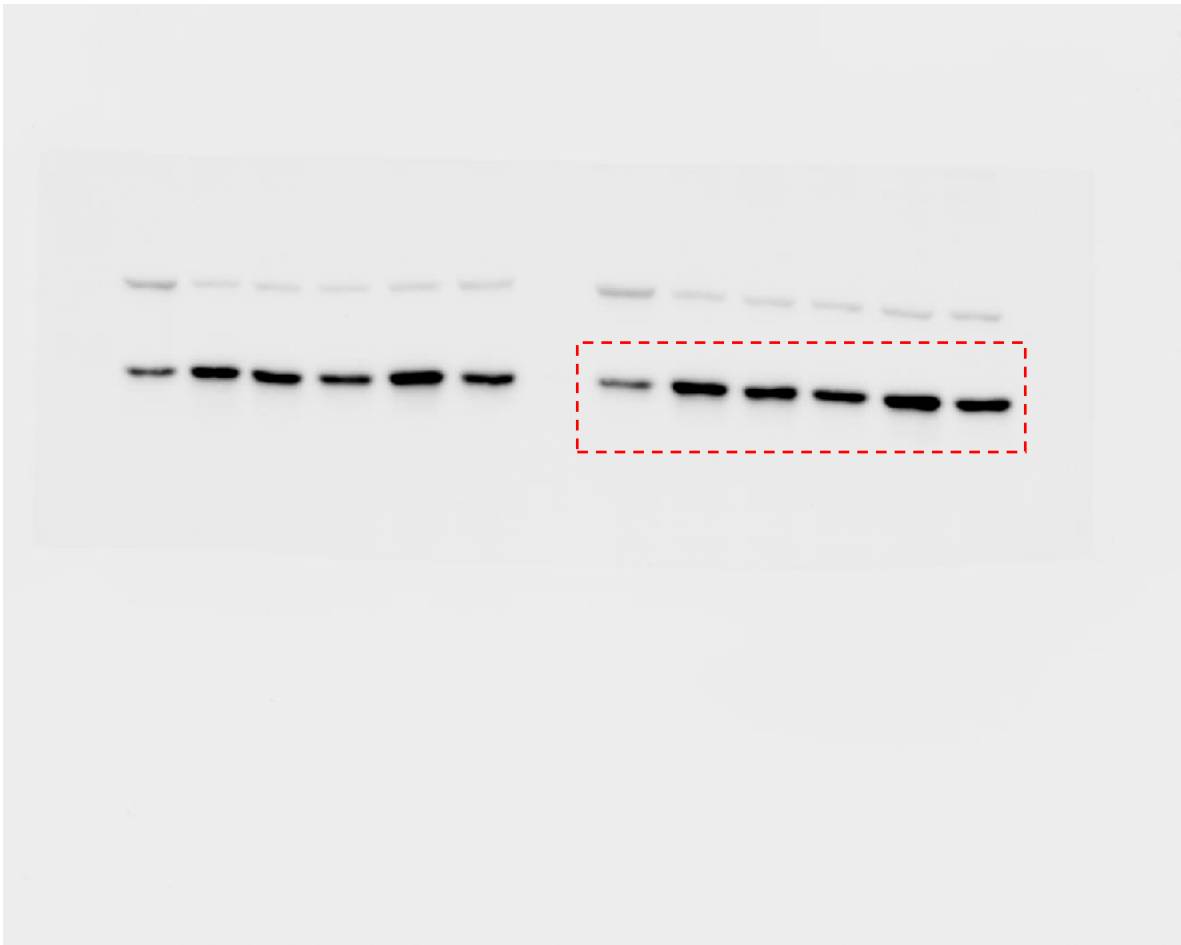

Supplement: Source Data Extended Data Fig. 4 — Unprocessed western blots. [file 41588_2022_1191_MOESM20_ESM.pdf]

p-FAK

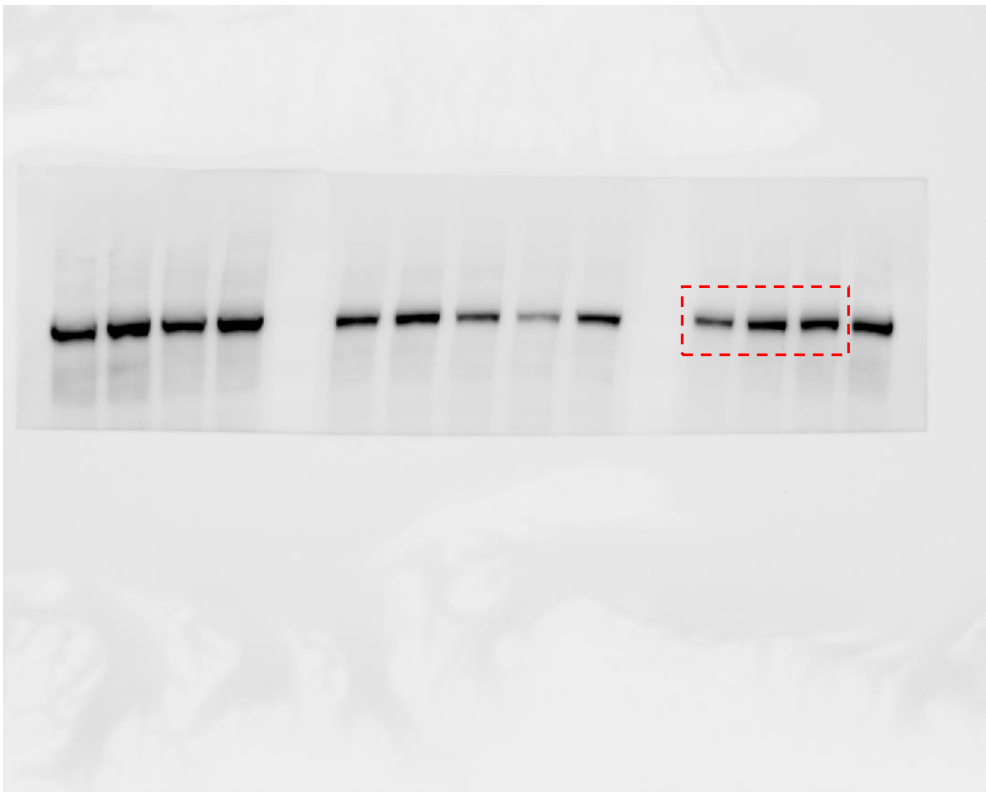

FAK

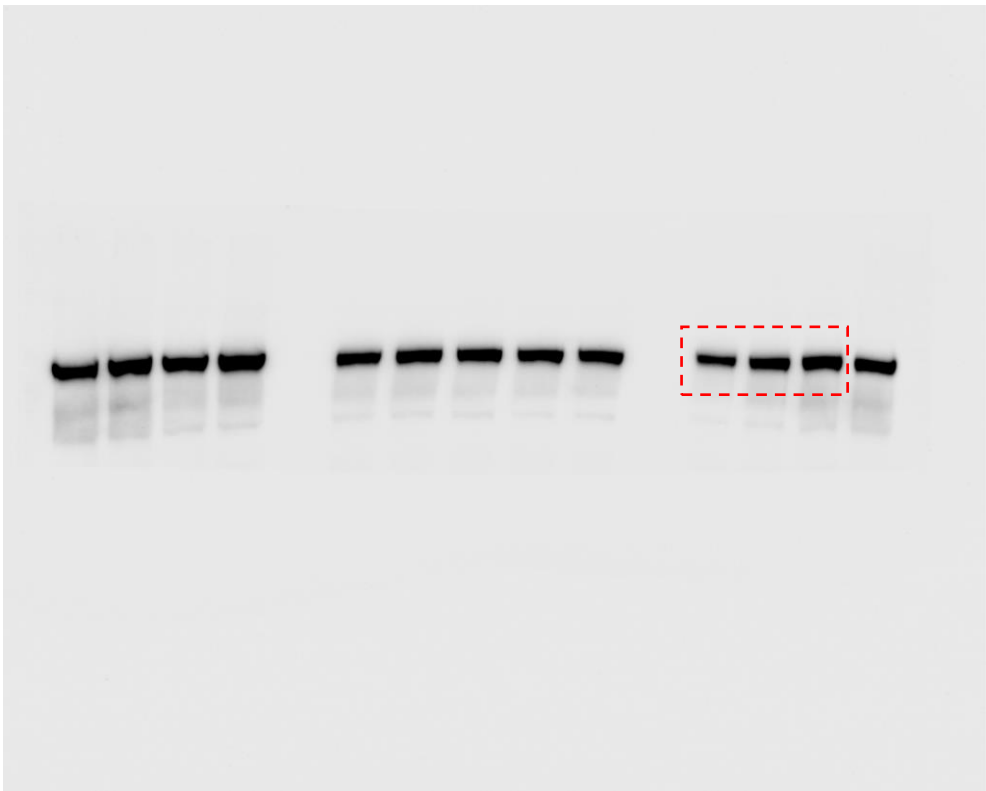

p-SRC

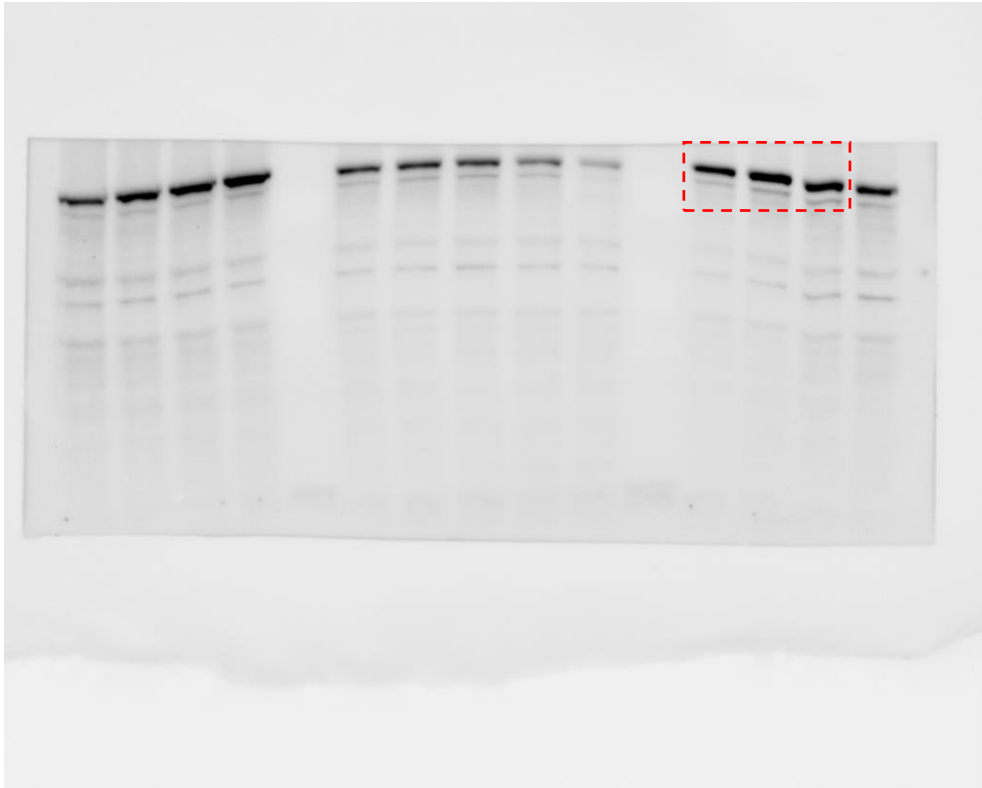

SRC

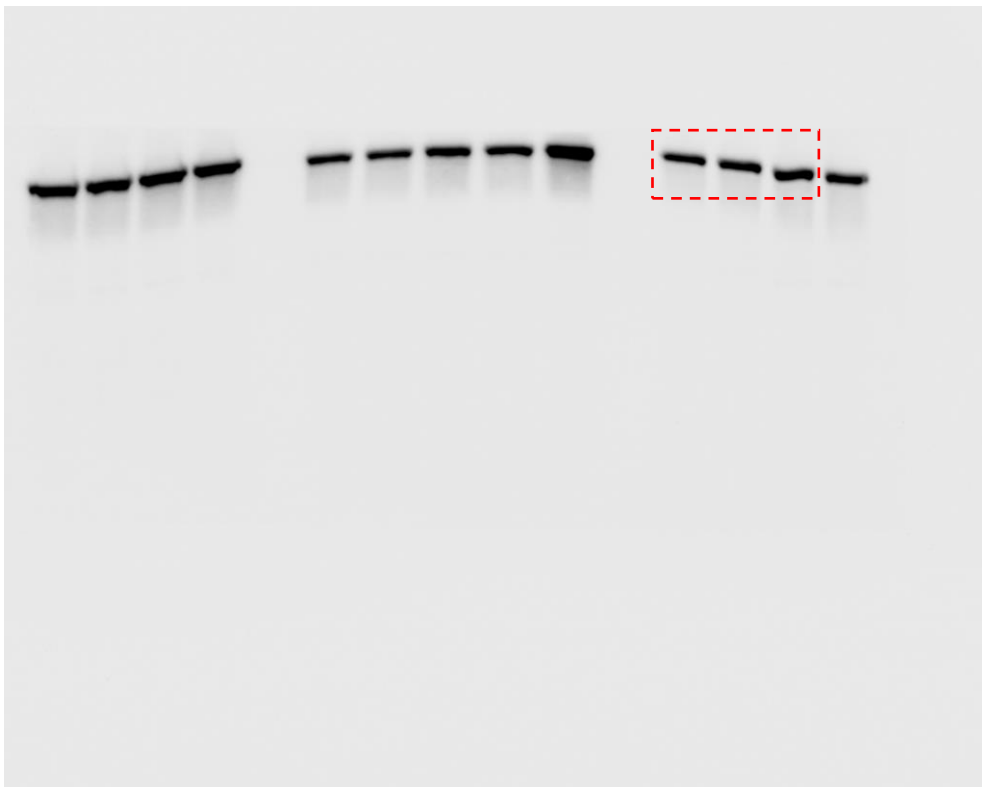

## Colorimetric

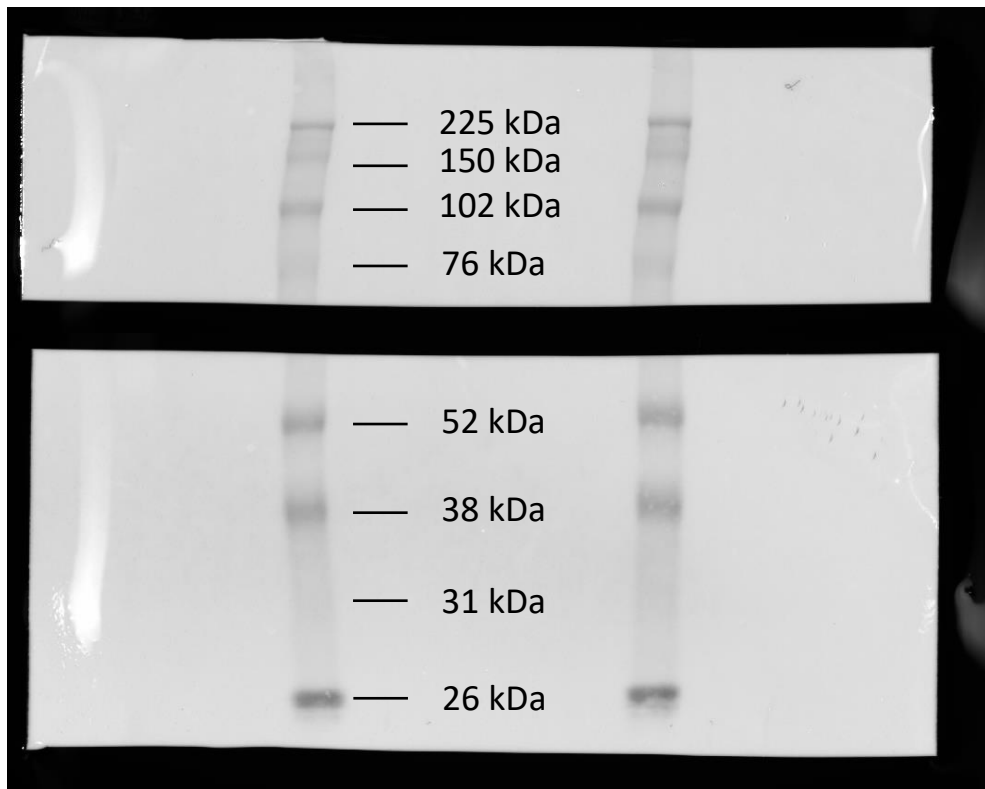

## GAPDH

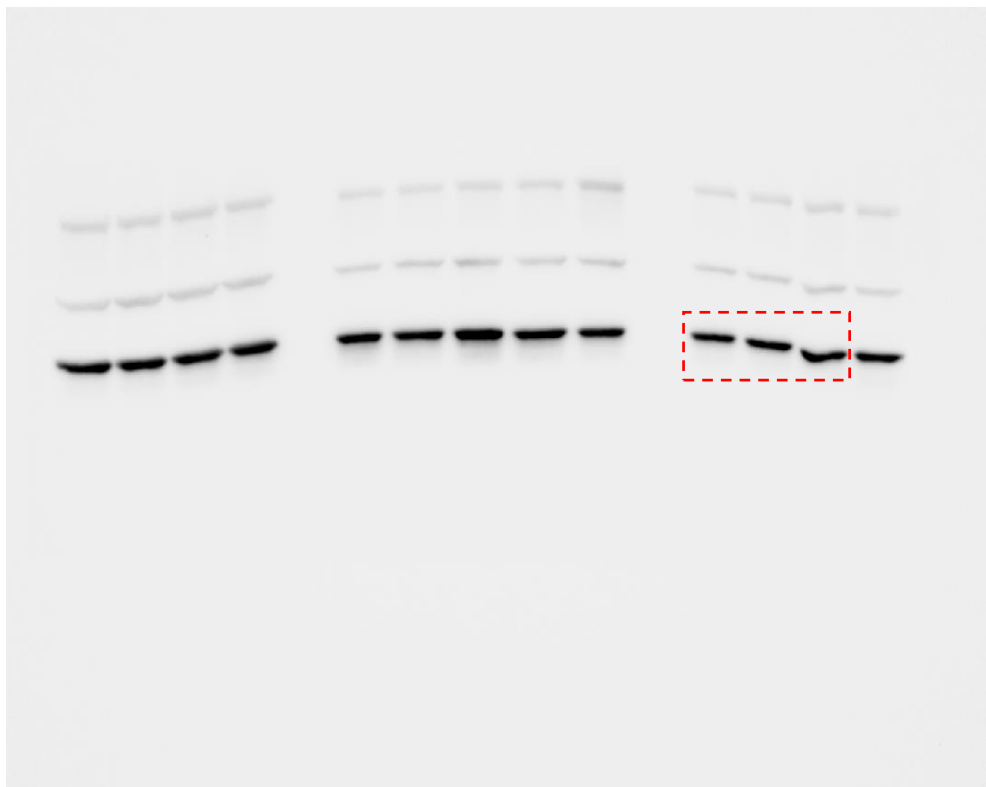

IGF1R

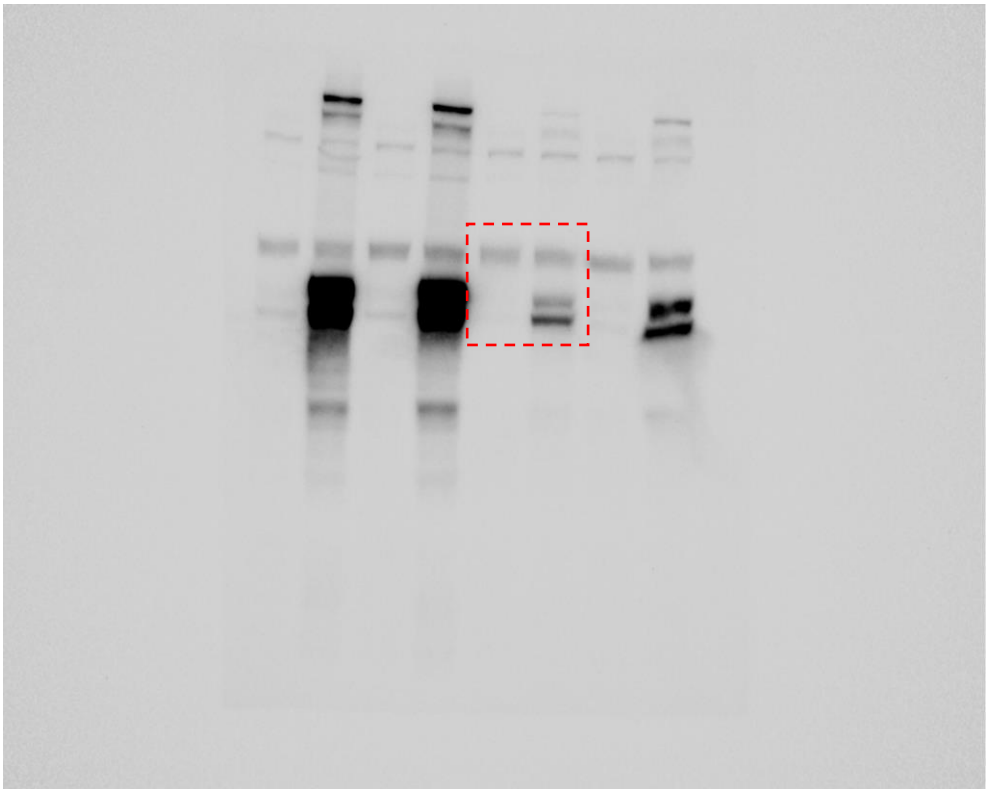

GAPDH

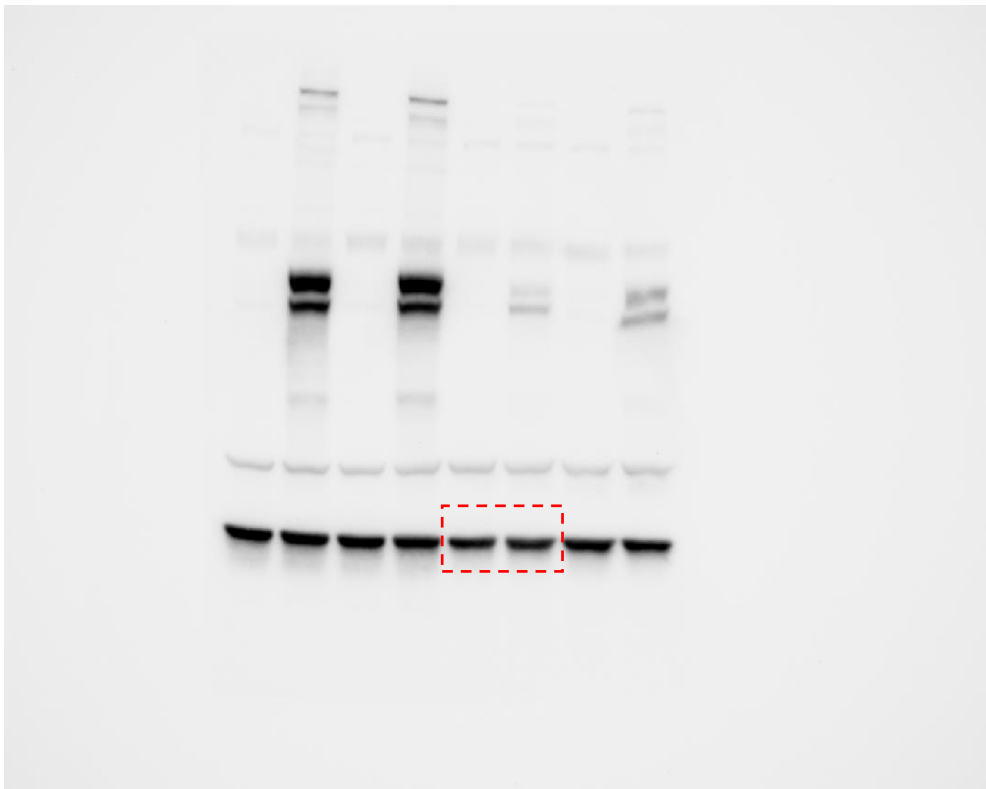

# Colorimetric

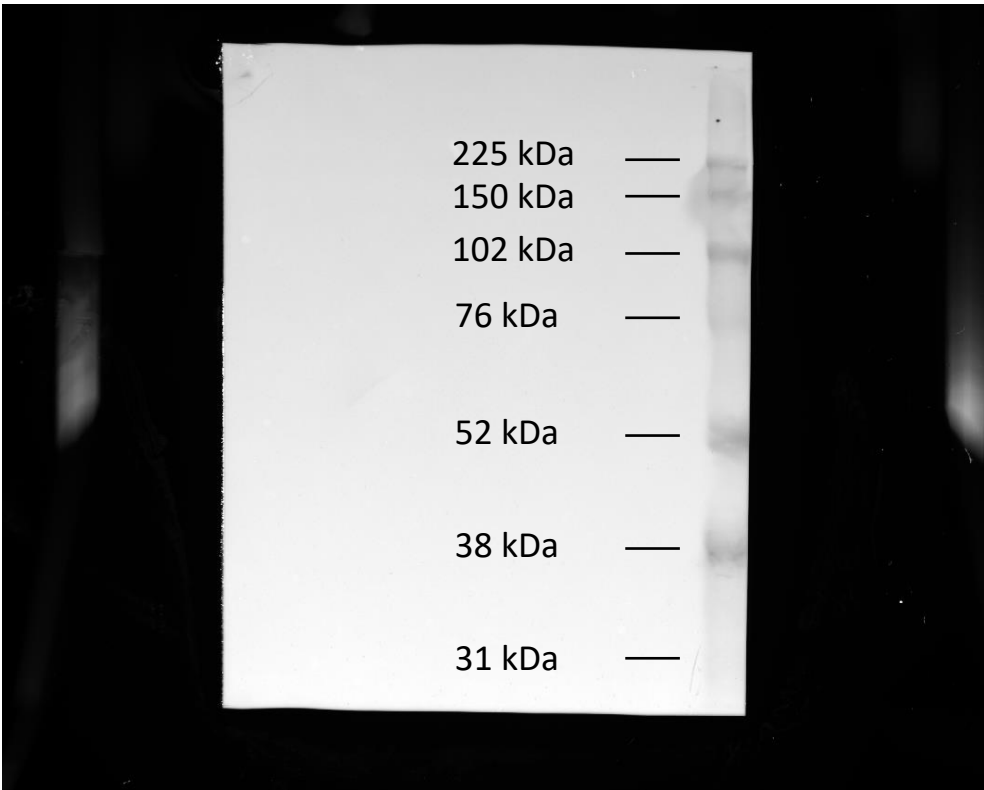

Supplement: Source Data Extended Data Fig. 9 — Unprocessed western blots. [file 41588_2022_1191_MOESM28_ESM.pdf]
