# Supplementary material for: Loss of NECTIN1 triggers melanoma dissemination upon local IGF1 depletion
Source: Nat Genet. 2022 Oct 13;54(12):1839–52. doi: 10.1038/s41588-022-01191-z (PMC9729115; doi:10.1038/s41588-022-01191-z)

Extended Data Fig 7e

FAK

SRC ►

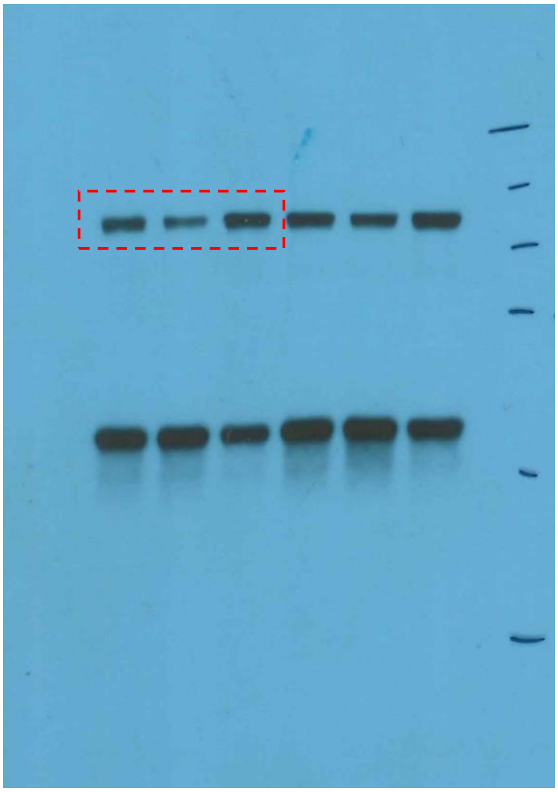

SRC

FAK ►

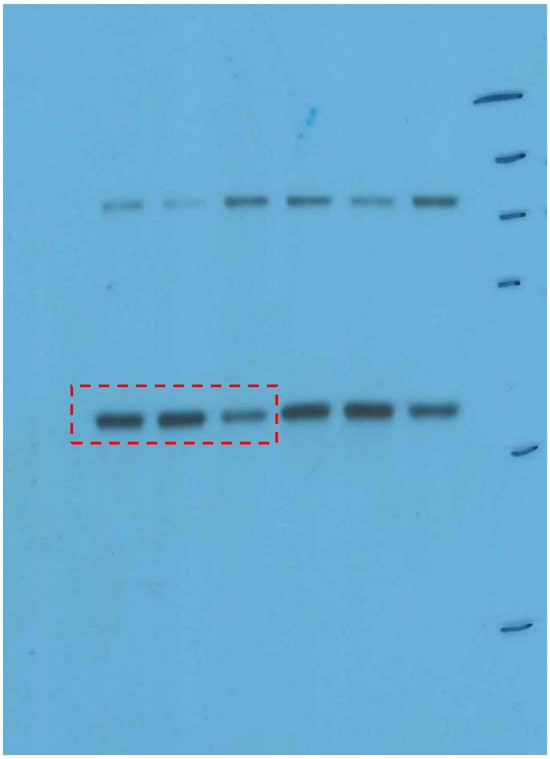

ACTIN

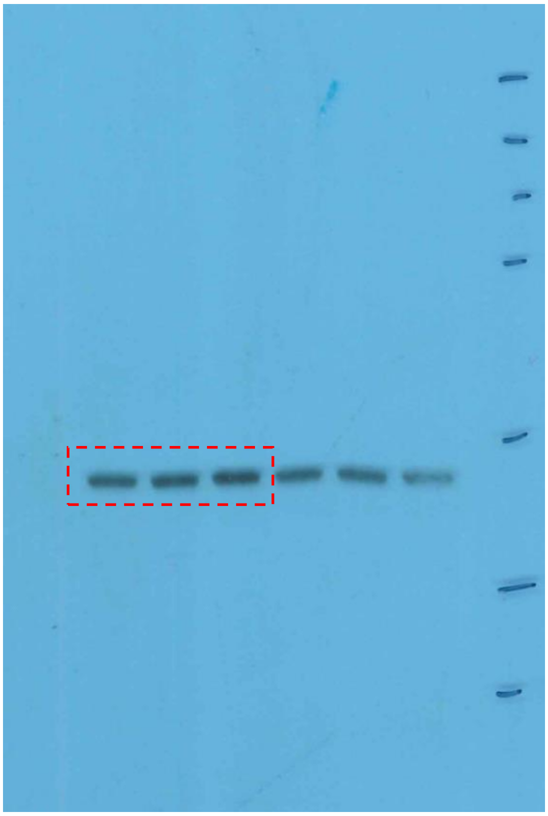

225 kDa
150 kDa
102 kDa
76 kDa
52 kDa
38 kDa
31 kDa

Extended Data Fig 7h

p-FAK

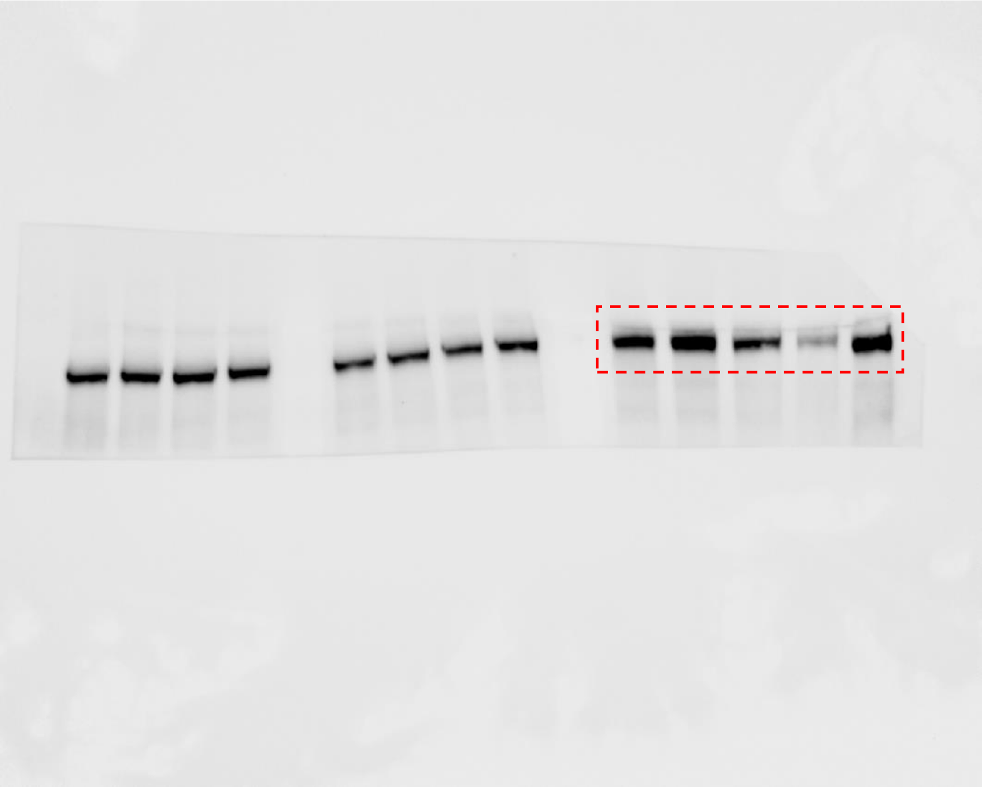

FAK

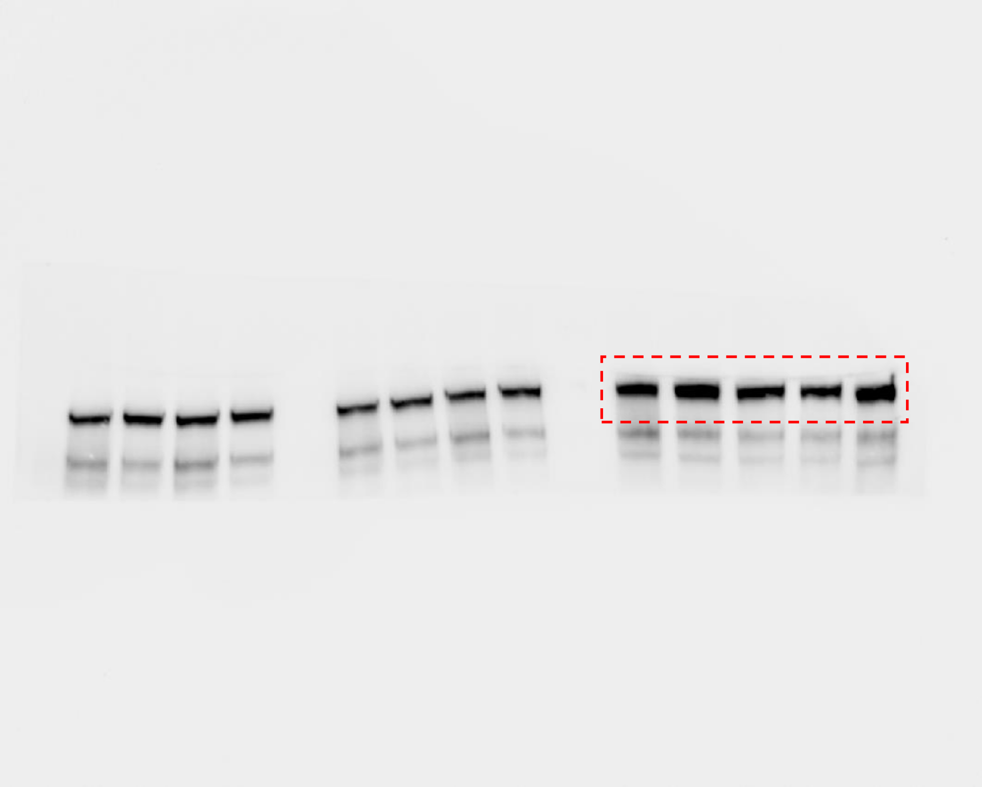

p-SRC

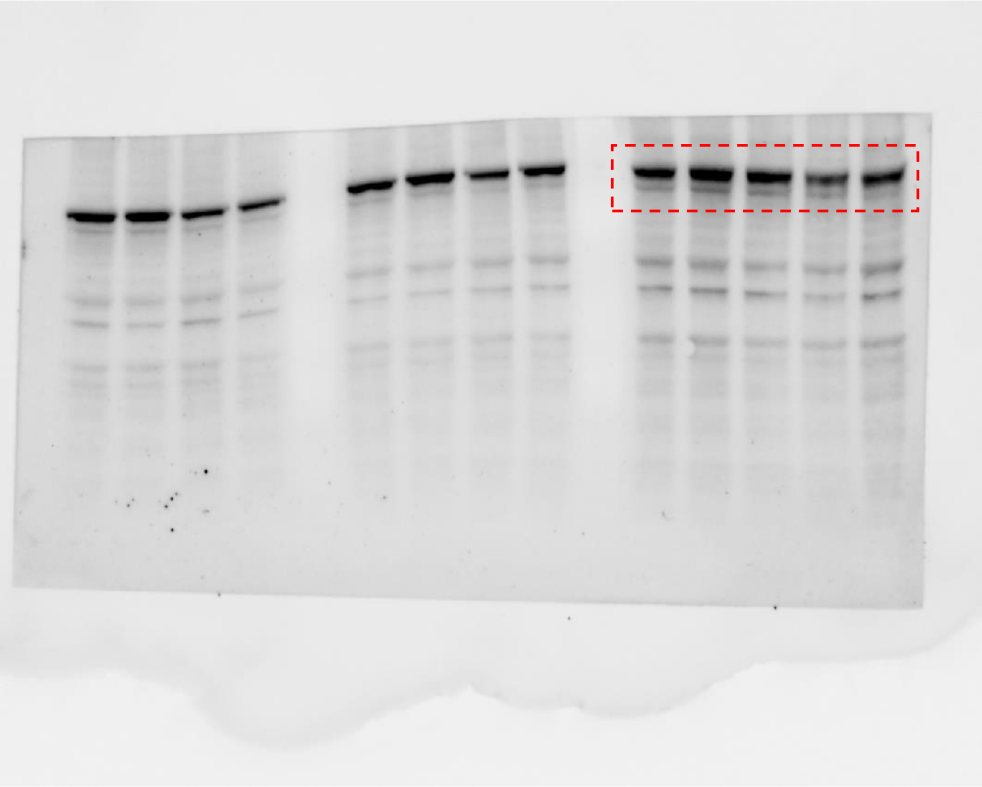

SRC

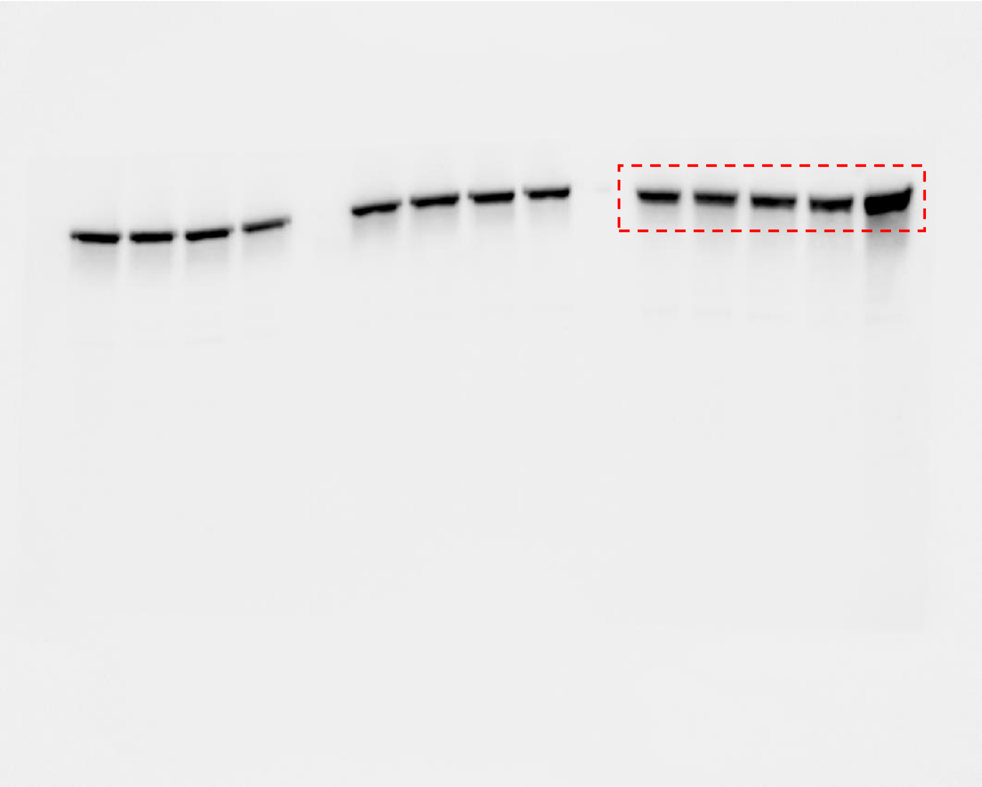

Colorimetric

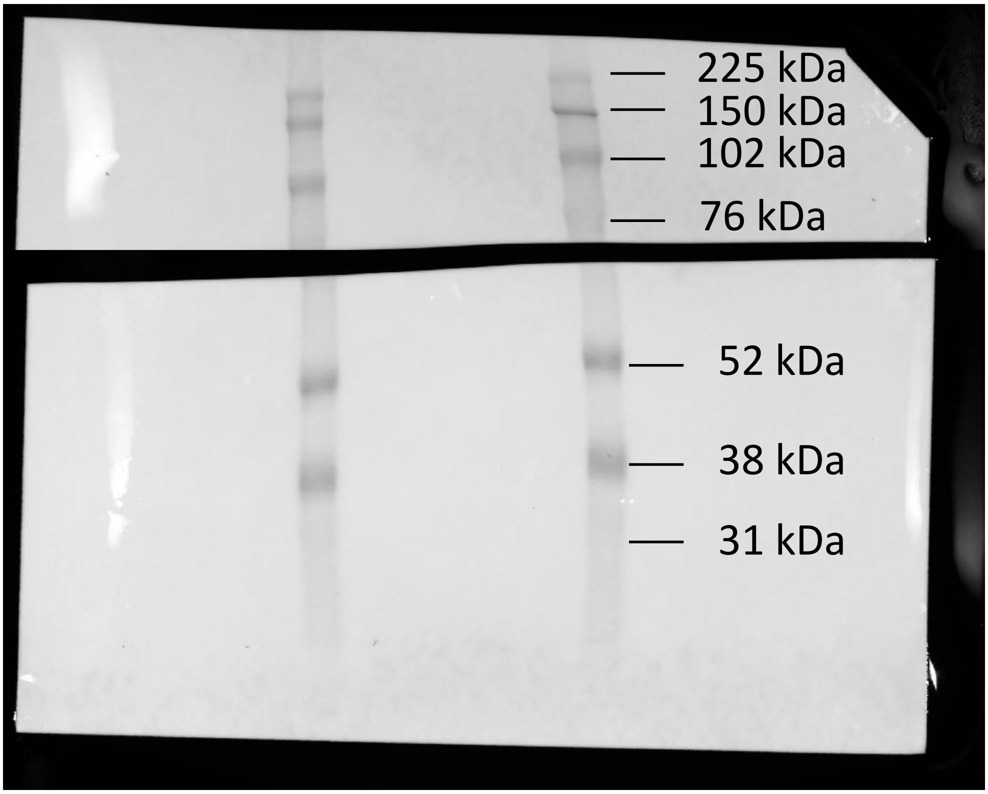

GAPDH

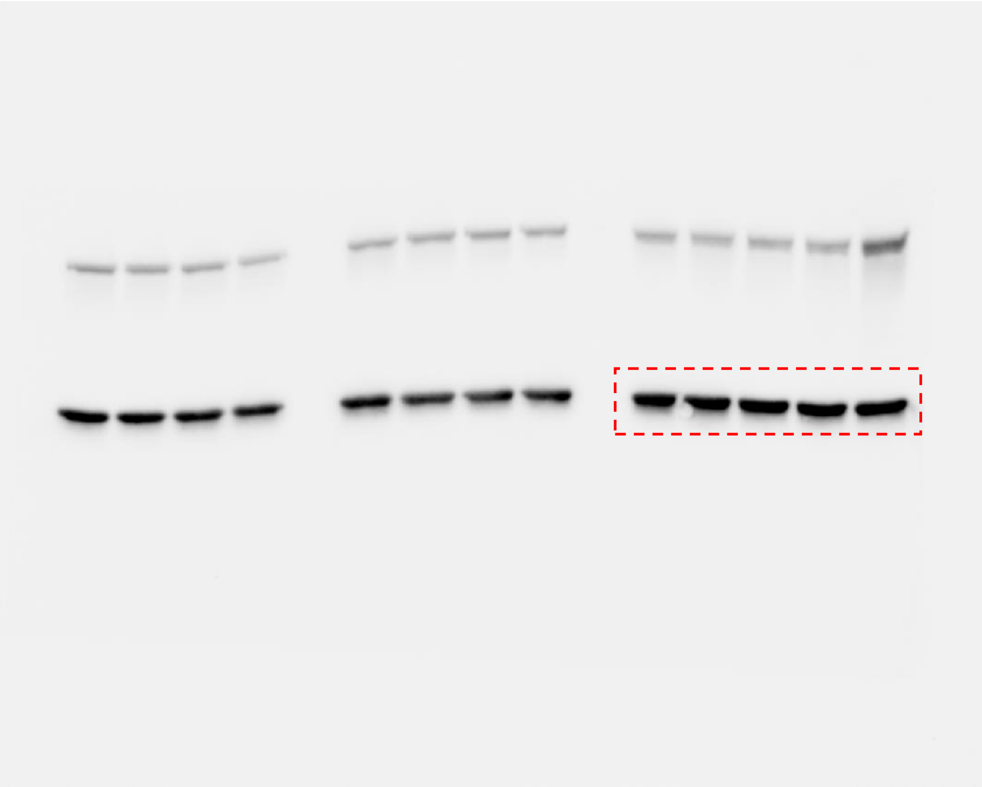

Supplement: Source Data Extended Data Fig. 7 — Unprocessed western blots. [file 41588_2022_1191_MOESM24_ESM.pdf]
