# Supplementary material for: Loss of NECTIN1 triggers melanoma dissemination upon local IGF1 depletion
Source: Nat Genet. 2022 Oct 13;54(12):1839–52. doi: 10.1038/s41588-022-01191-z (PMC9729115; doi:10.1038/s41588-022-01191-z)
Supplement: Source Data Extended Data Fig. 8 — Unprocessed western blots. [file 41588_2022_1191_MOESM26_ESM.pdf]

Extended Data Fig 8g

NECTIN1

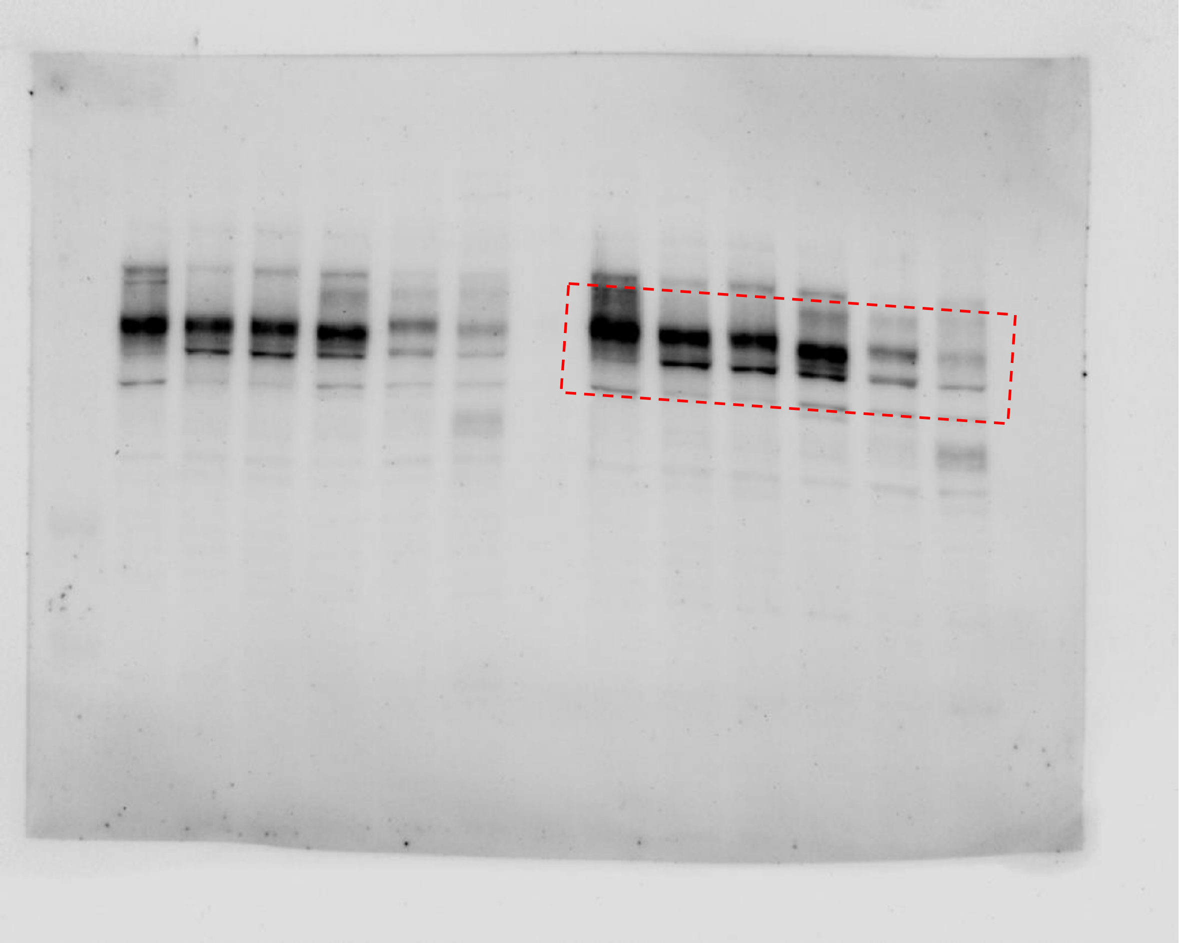

GAPDH

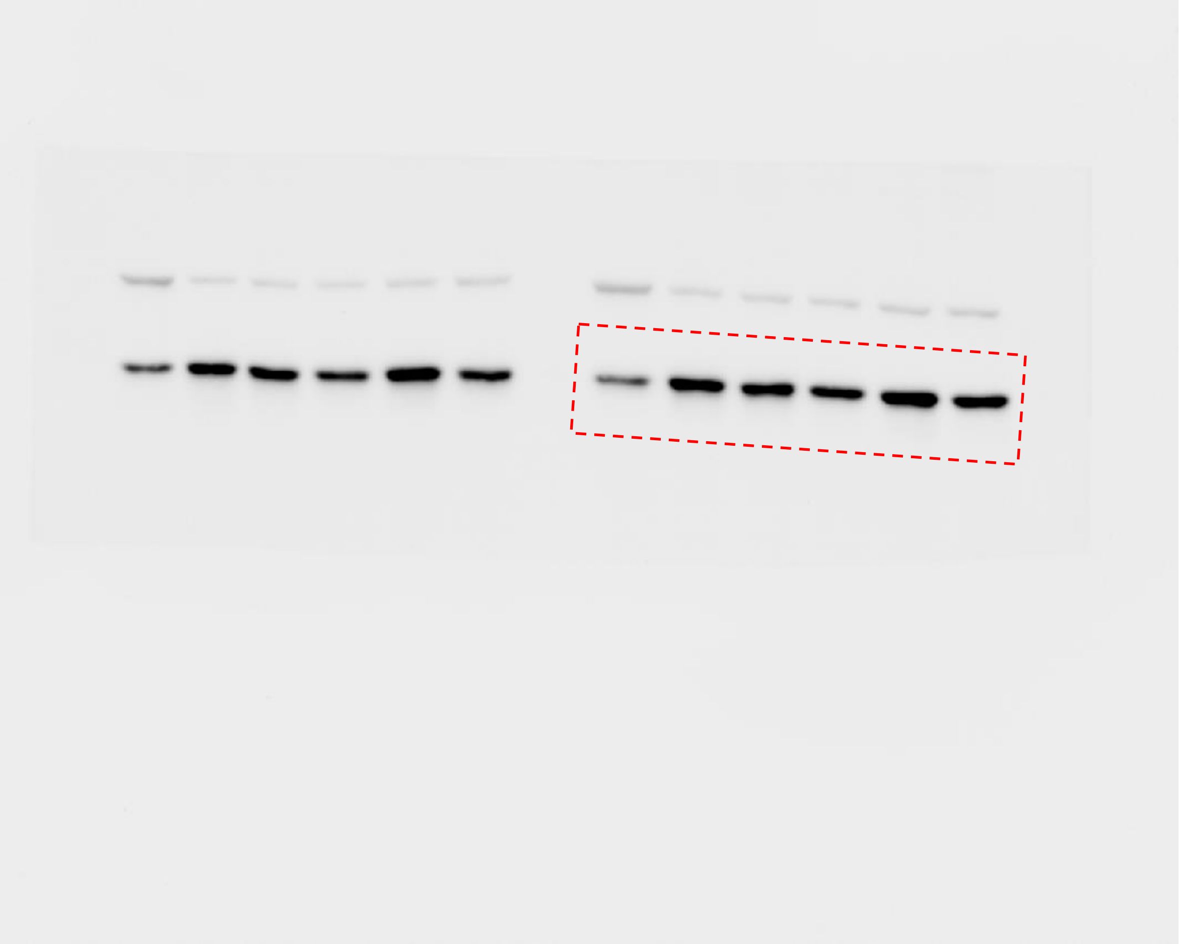

p-FAK

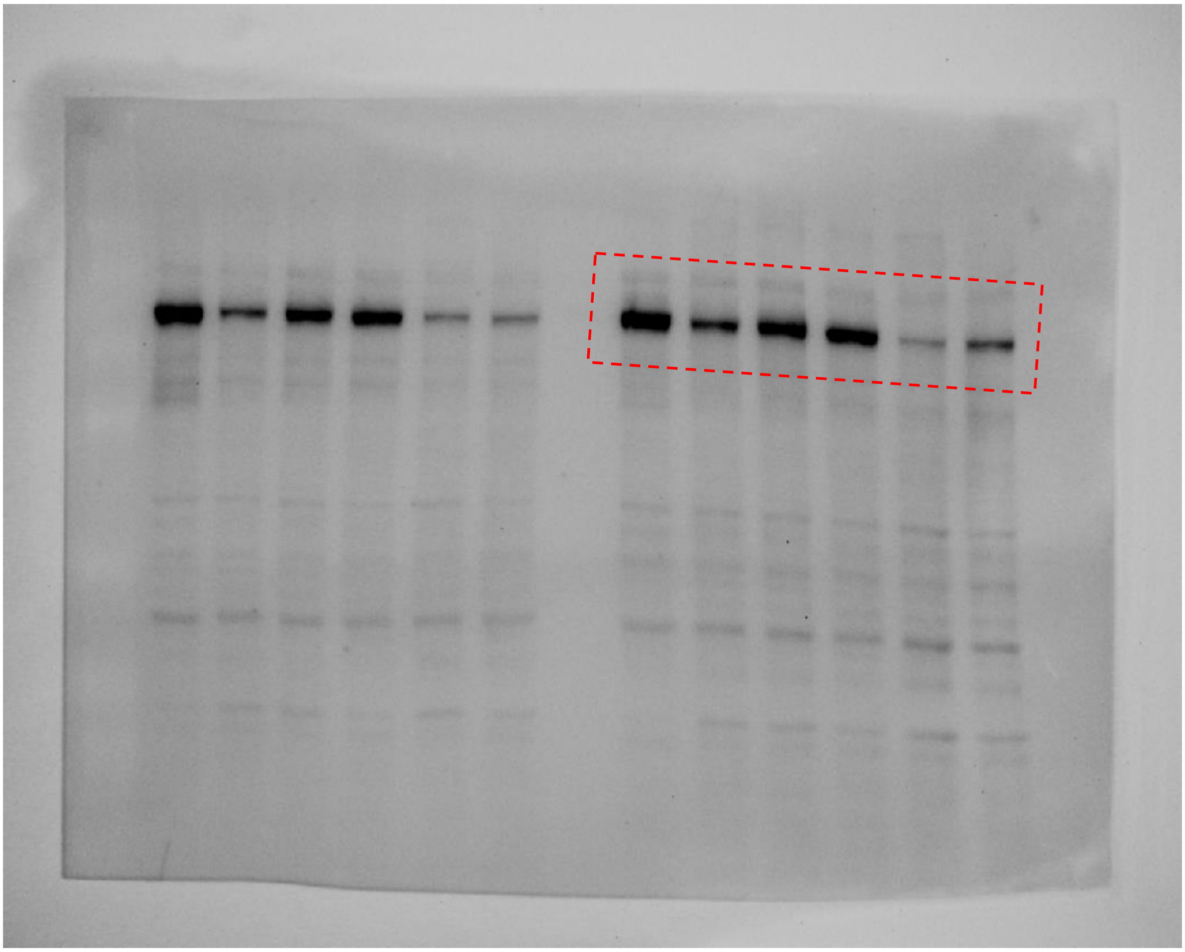

FAK

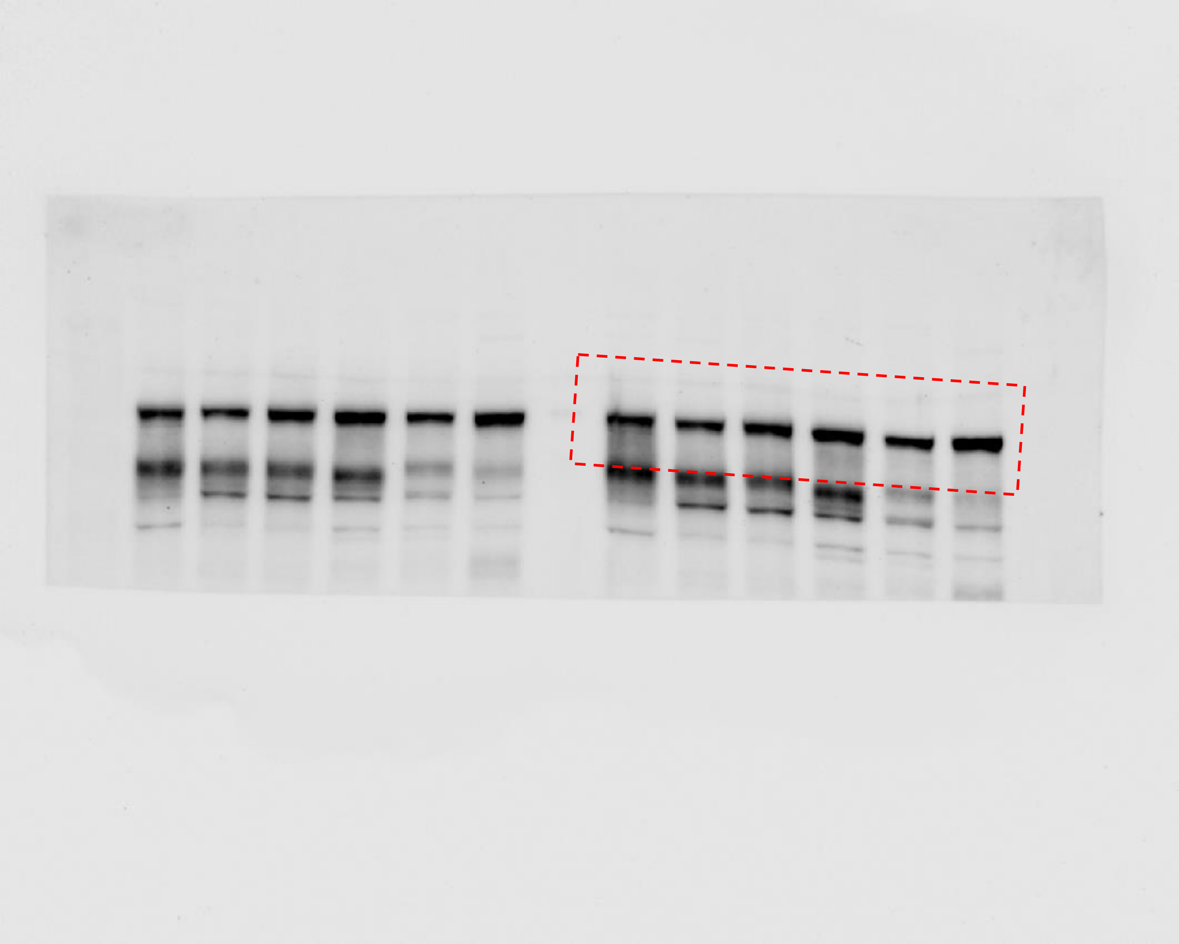

Colorimetric

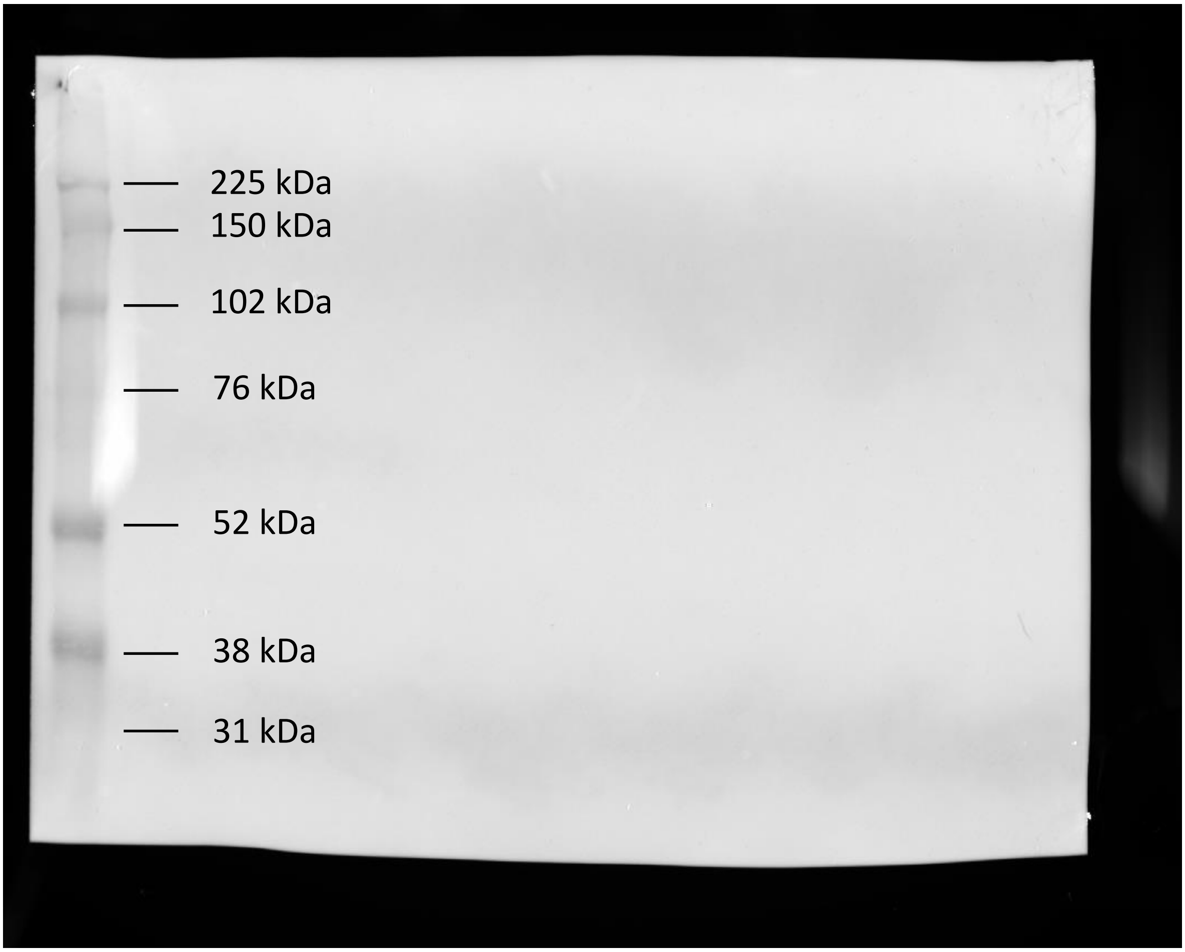

Extended Data Fig 8i

p-FAK

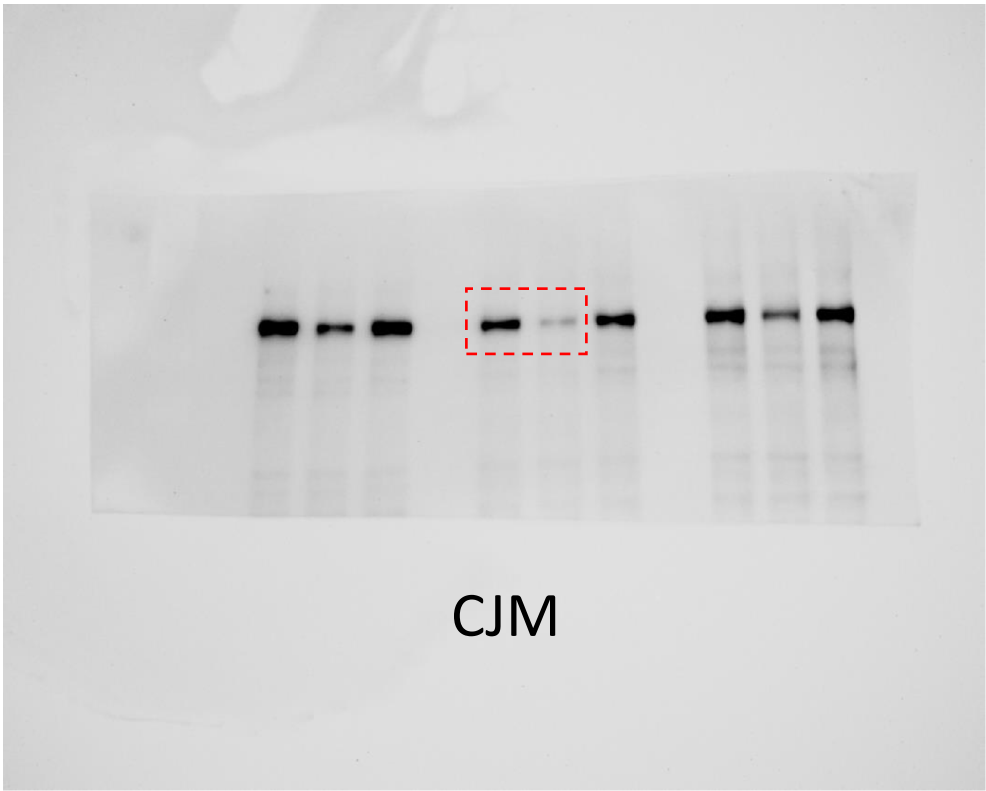

FAK

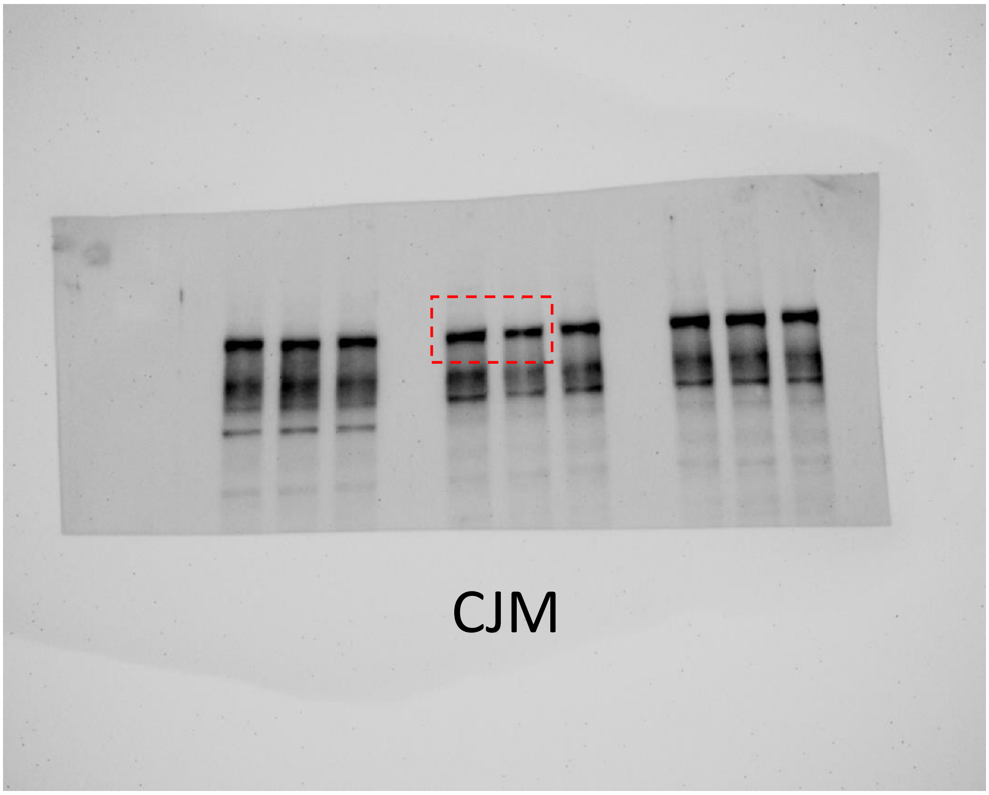

NECTIN1

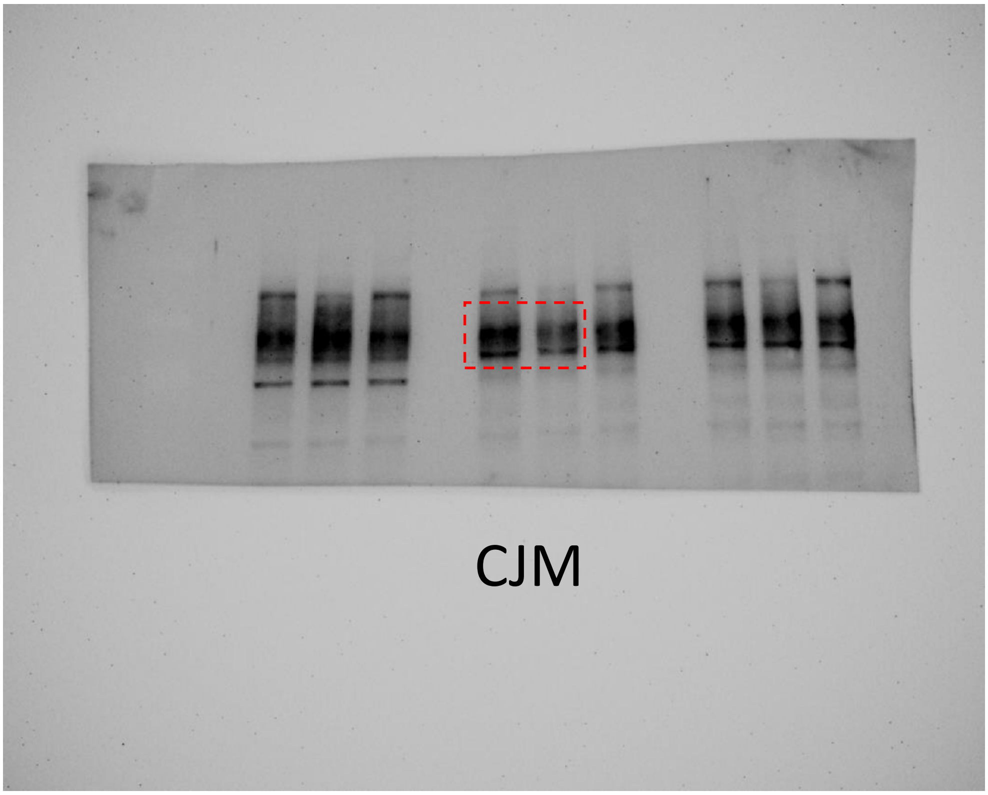

GAPDH

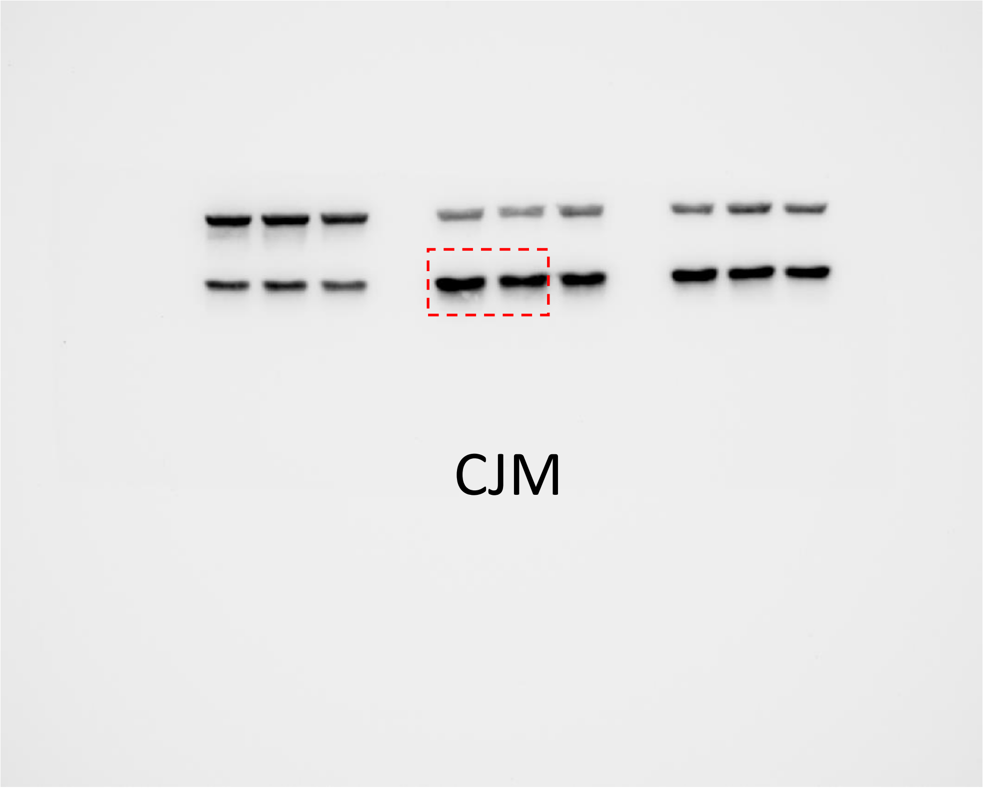

Colorimetric

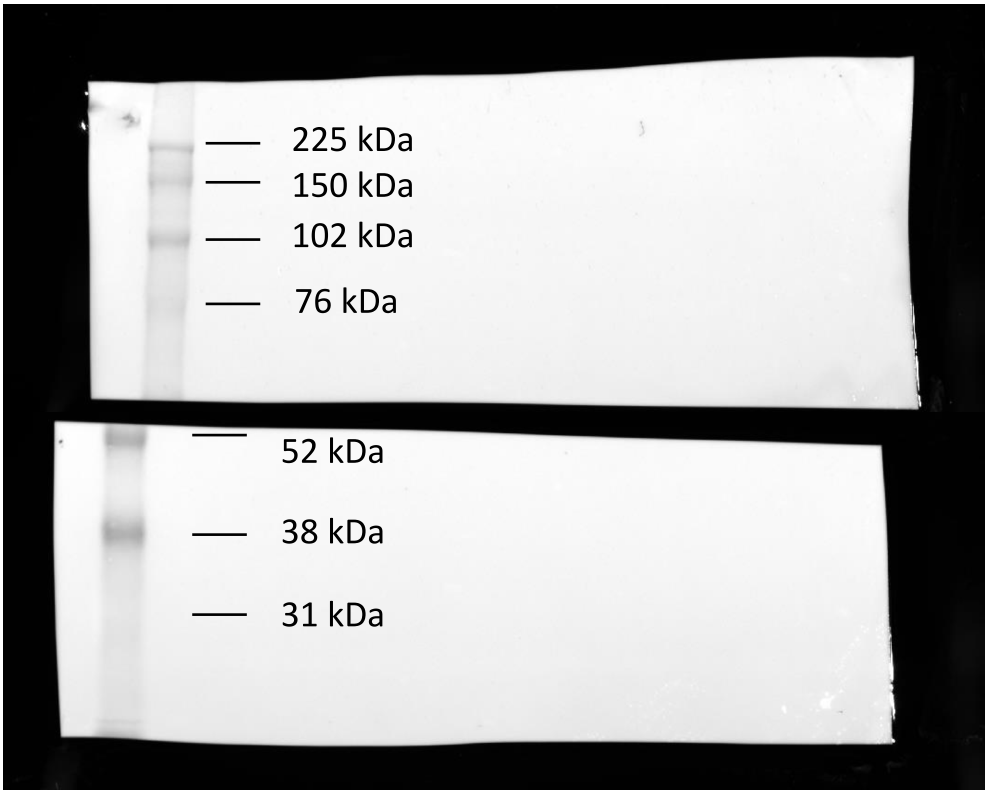

CJM

p-FAK

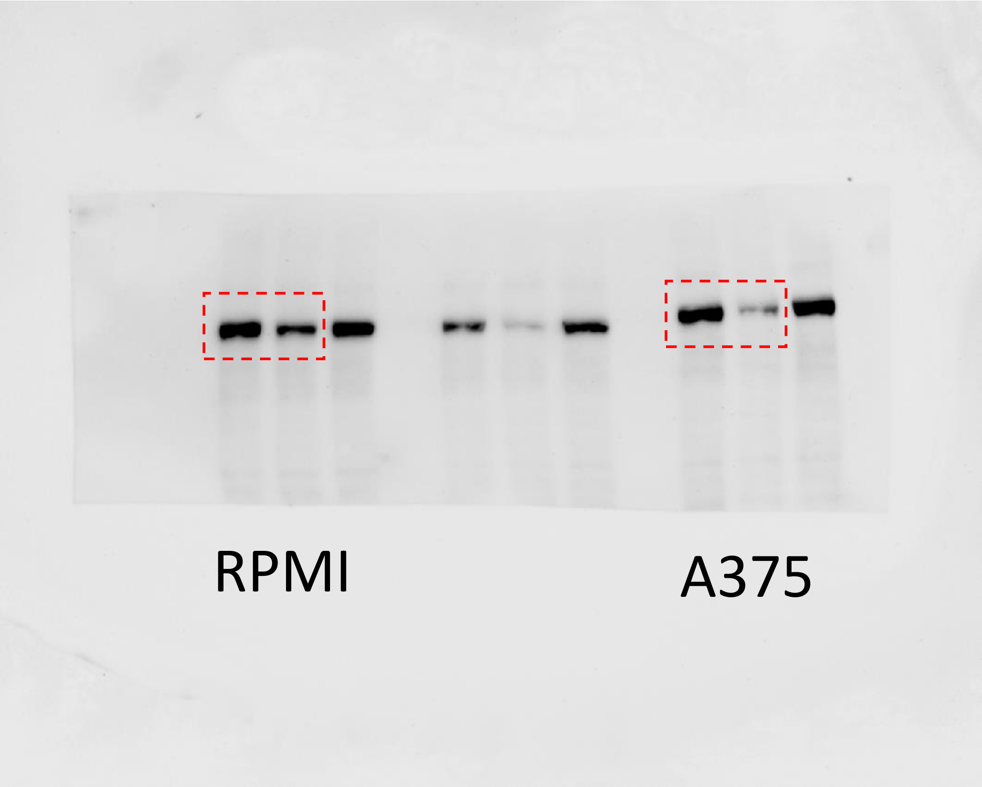

FAK

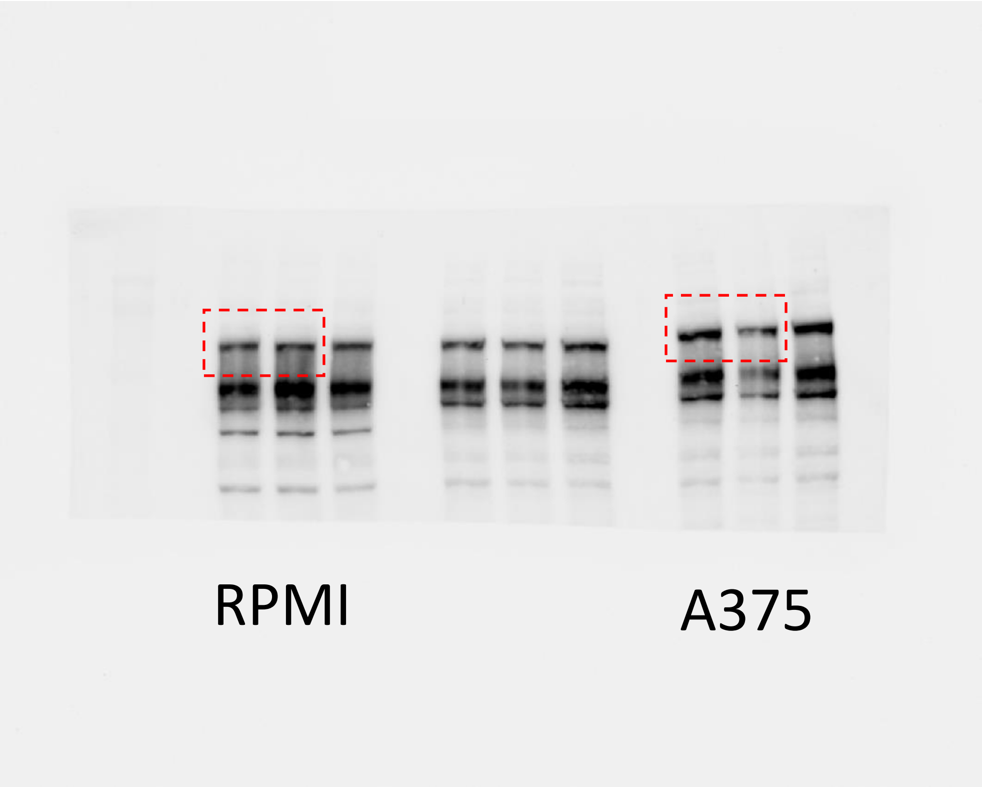

NECTIN1

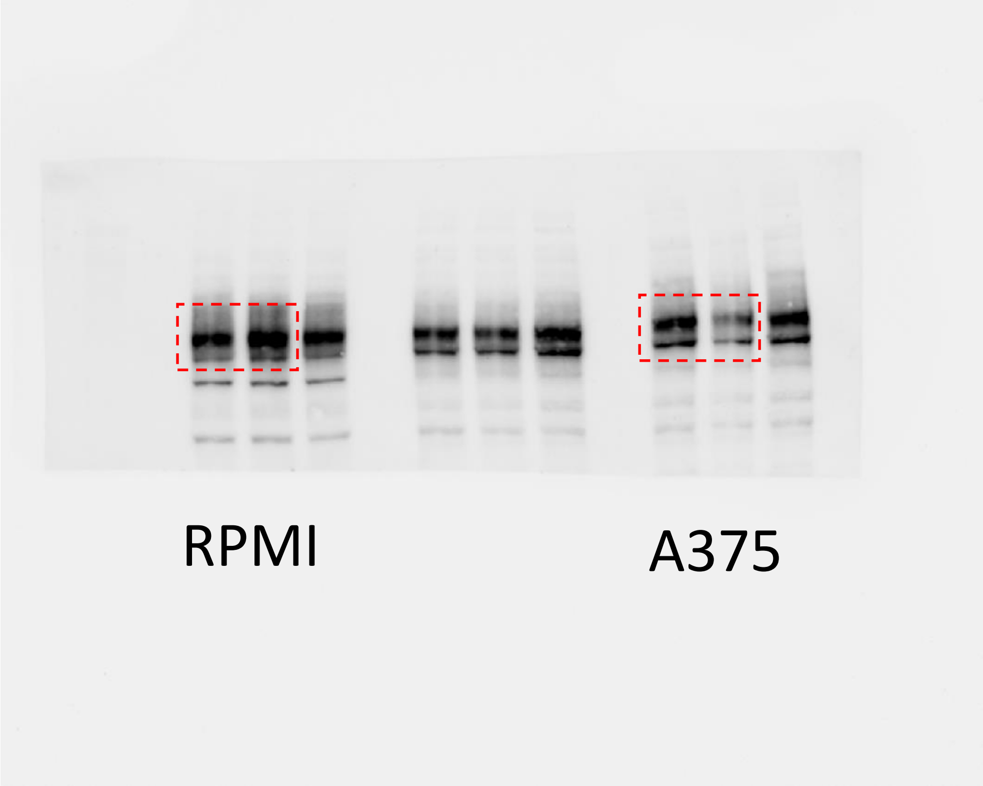

GAPDH

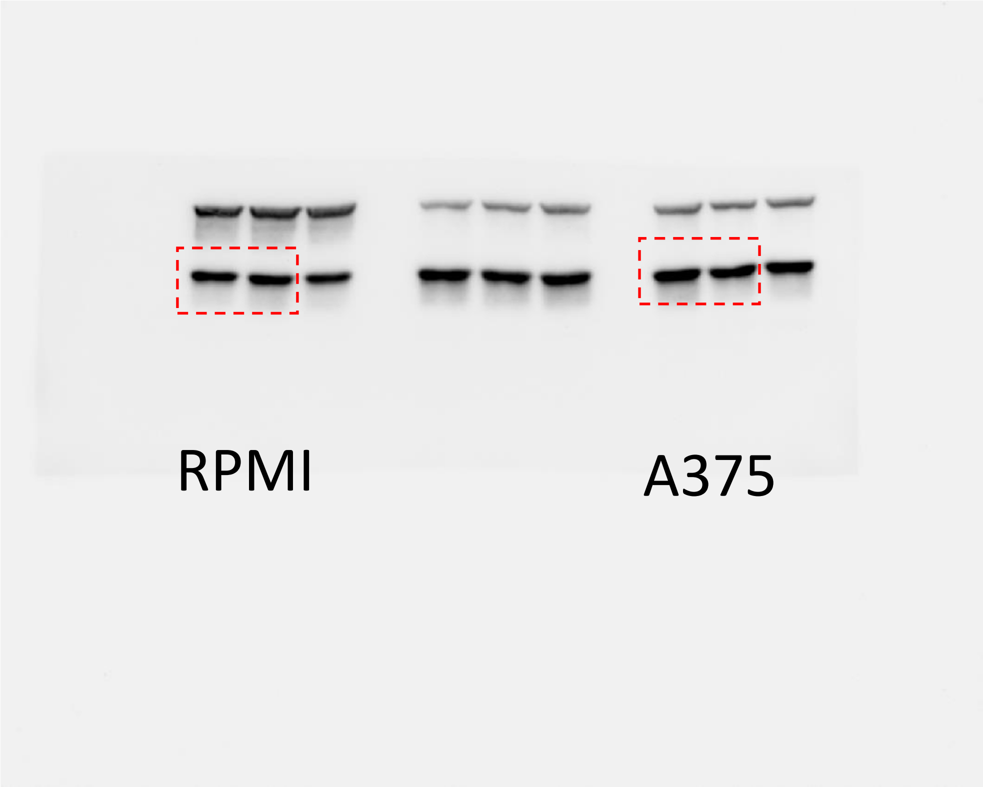

Colorimetric

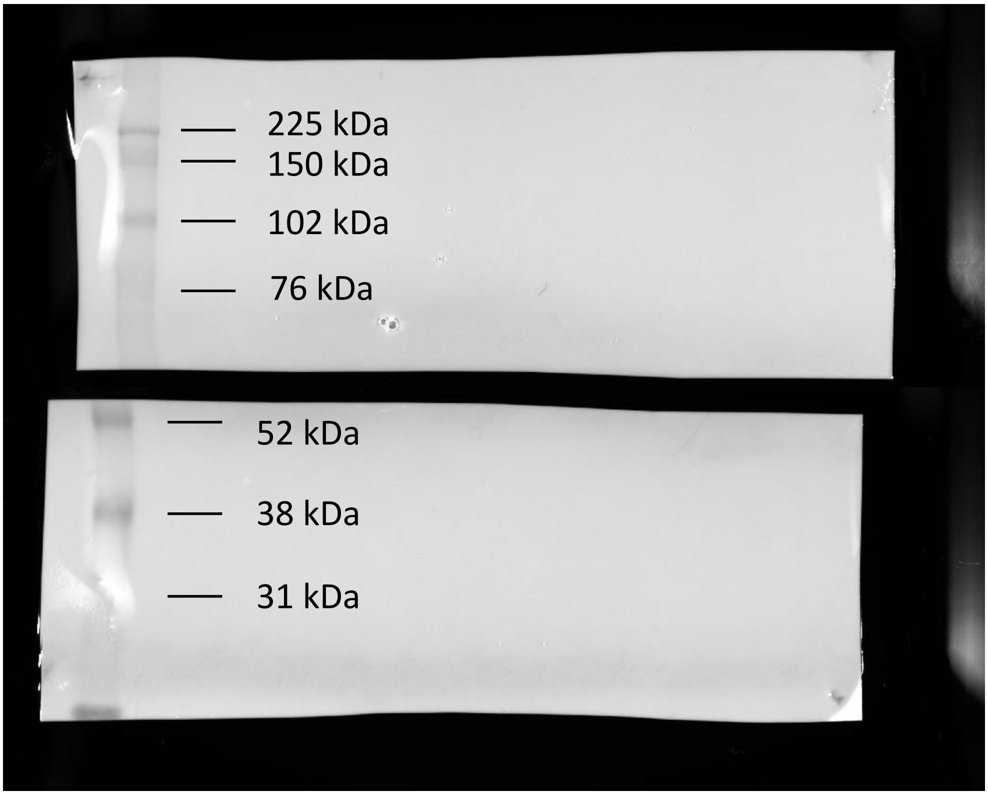

RPMI A375

p-FAK

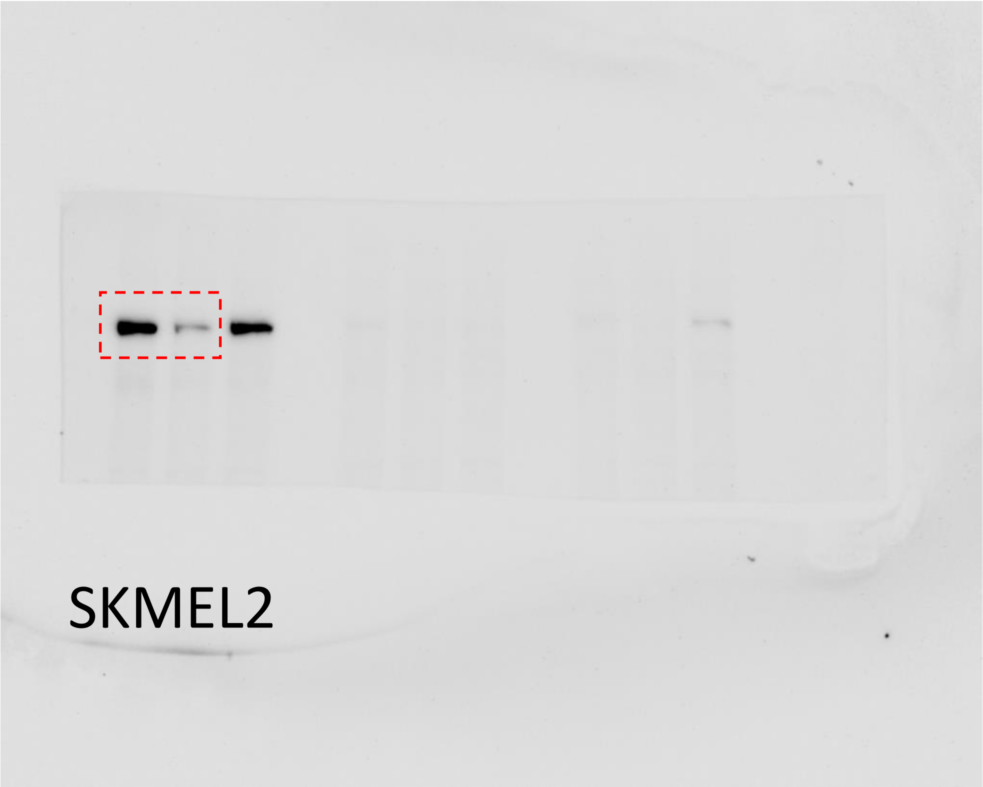

FAK

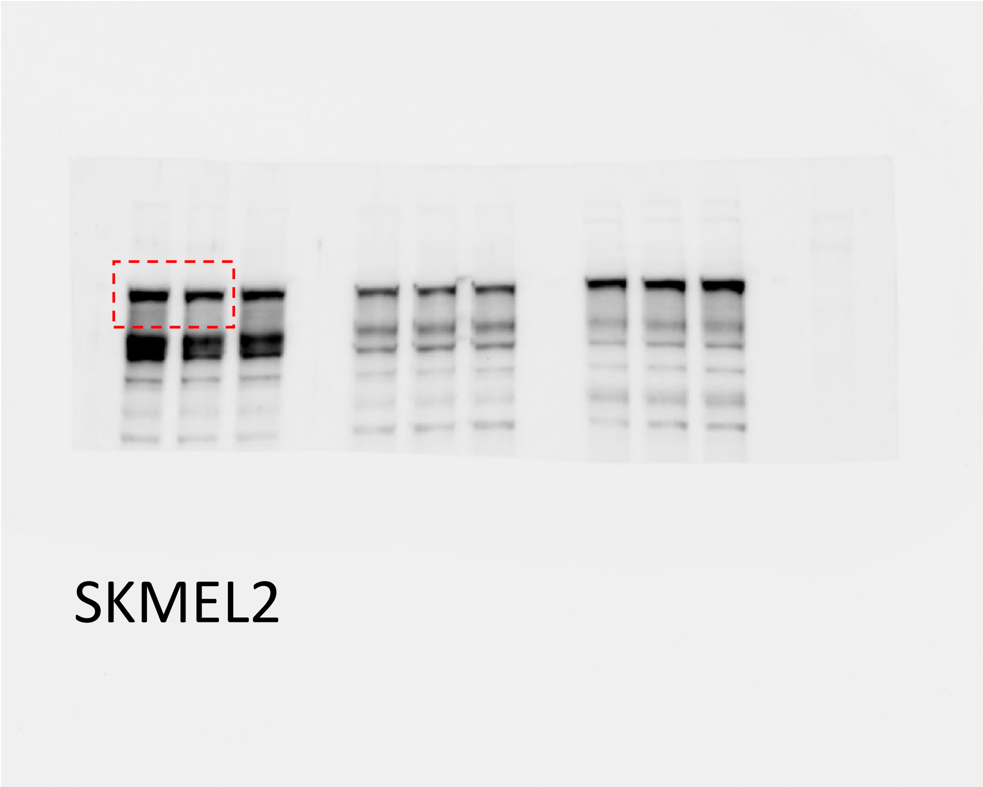

NECTIN1

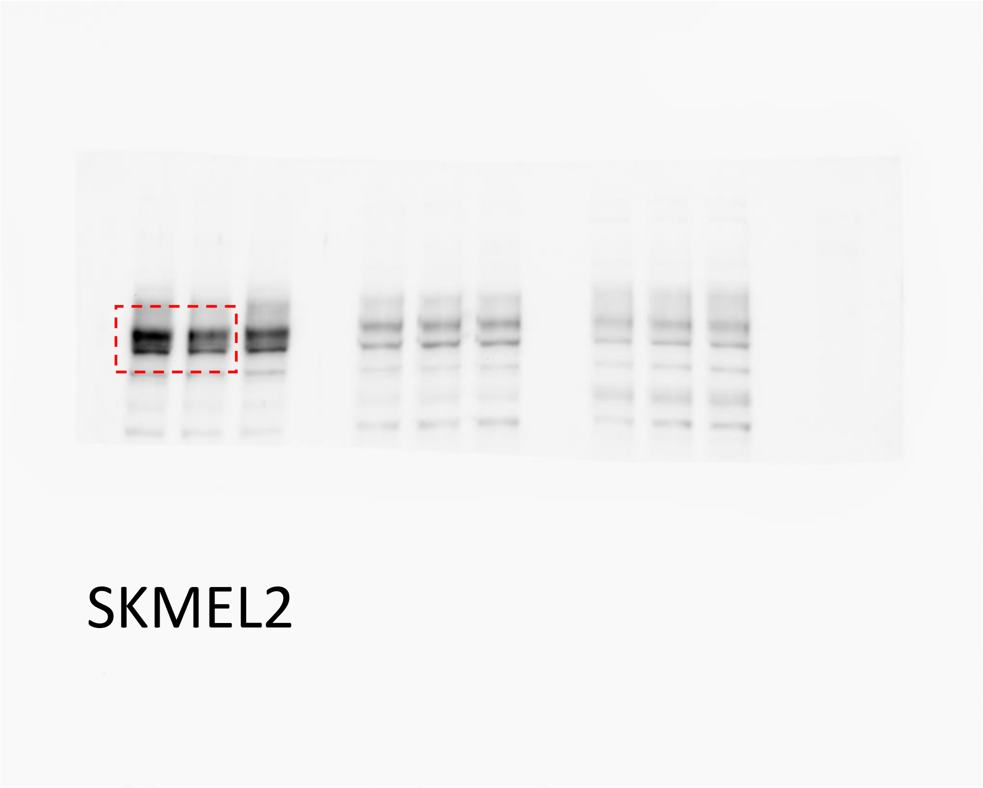

GAPDH

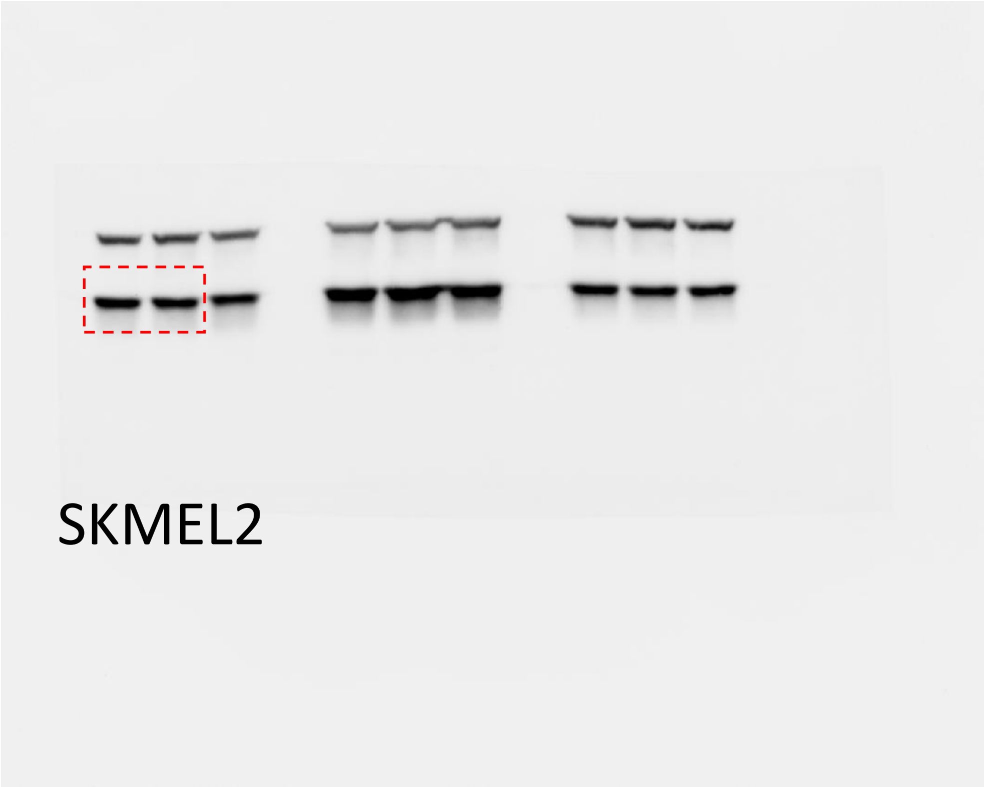

Colorimetric

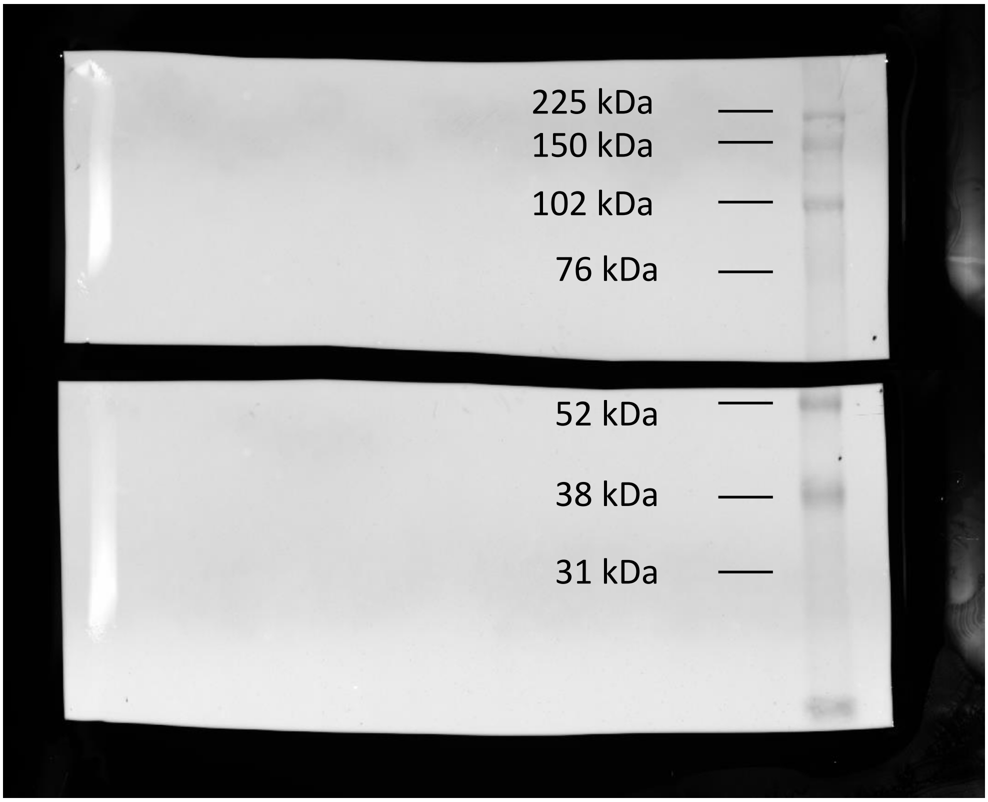

SKMEL2
